# Human HELB is a processive motor protein that catalyzes RPA clearance from single-stranded DNA

**DOI:** 10.1073/pnas.2112376119

**Published:** 2022-04-06

**Authors:** Silvia Hormeno, Oliver J. Wilkinson, Clara Aicart-Ramos, Sahiti Kuppa, Edwin Antony, Mark S. Dillingham, Fernando Moreno-Herrero

**Affiliations:** ^a^Department of Macromolecular Structures, Centro Nacional de Biotecnología, Consejo Superior de Investigaciones Científicas, 28049 Madrid, Spain;; ^b^DNA:Protein Interactions Unit, School of Biochemistry, University of Bristol, Bristol BS8 1TD, United Kingdom;; ^c^Department of Biochemistry, Saint Louis University, St. Louis, MO 63104

**Keywords:** HELB, RPA, DNA replication, repair, single-molecule experiments

## Abstract

Human DNA helicase B (HELB) is a poorly characterized helicase suggested to play both positive and negative regulatory roles in DNA replication and recombination. In this work, we used bulk and single-molecule approaches to characterize the biochemical activities of HELB protein with a particular focus on its interactions with Replication Protein A (RPA) and RPA–single-stranded DNA (ssDNA) filaments. HELB is a monomeric protein that binds tightly to ssDNA with a site size of ∼20 nucleotides. It couples ATP hydrolysis to translocation along ssDNA in the 5′ to 3′ direction accompanied by the formation of DNA loops. HELB also displays classical helicase activity, but this is very weak in the absence of an assisting force. HELB binds specifically to human RPA, which enhances its ATPase and ssDNA translocase activities but inhibits DNA unwinding. Direct observation of HELB on RPA nucleoprotein filaments shows that translocating HELB concomitantly clears RPA from ssDNA. This activity, which can allow other proteins access to ssDNA intermediates despite their shielding by RPA, may underpin the diverse roles of HELB in cellular DNA transactions.

## Significance

Single-stranded DNA (ssDNA) is a key intermediate in many cellular DNA transactions, including DNA replication, repair, and recombination. Nascent ssDNA is rapidly bound by the Replication Protein A (RPA) complex, forming a nucleoprotein filament that both stabilizes ssDNA and mediates downstream processing events. Paradoxically, however, the very high affinity of RPA for ssDNA may block the recruitment of further factors. In this work, we show that RPA–ssDNA nucleoprotein filaments are specifically targeted by the human HELB helicase. Recruitment of HELB by RPA–ssDNA activates HELB translocation activity, leading to processive removal of upstream RPA complexes. This RPA clearance activity may underpin the diverse roles of HELB in replication and recombination.

The human HELB protein was first identified as a homolog of a putative murine replicative helicase (1–3). Since then, various functions have been assigned to the protein, including a role in the onset of chromosomal DNA replication (2), cellular recovery from replication stress (4), promotion of Cdc45 chromatin binding (5), resolution of DNA secondary CGG nucleotides repeat structures (6), and stimulation of RAD51-mediated 5′–3′ heteroduplex extension to promote homologous recombination (HR) (7). Most recently and in apparent contradiction to the role in the stimulation of HR, HELB was proposed to inhibit homology-dependent double-stranded DNA break (DSB) repair by antagonizing the processive resection nucleases EXO1 and DNA2/BLM during the G0/G1 phases of the cell cycle (8). In agreement with this idea, HELB forms nuclear foci in response to DNA damage and is phosphorylated by cyclin-dependent kinase (CDK), causing localization to the nucleus in G1 and to the cytoplasm during S/G2. The formation of HELB damage foci is dependent on the main eukaryotic single-stranded DNA (ssDNA) binding protein Replication Protein A (RPA) (9), which has been shown to interact physically with HELB (4, 8). Although the interaction with RPA is potentially critical for all putative functions of HELB, the ability of this motor protein to modulate the formation, remodeling, or removal of RPA nucleoprotein filaments has never been studied and is the focus of the work presented here.

The filaments formed between RPA and ssDNA are critical intermediates in DNA replication, recombination, and repair (10–12). RPA not only shields ssDNA from nucleolytic degradation, but it is also involved in the recruitment or exclusion of other factors from ssDNA, the regulation of DNA replication and repair, and the initiation of cell signaling cues that link these pathways to the cell cycle and its progression through checkpoints (13). Interestingly, many helicases and helicase-like proteins share intimate physical and functional interactions with ssDNA binding proteins (14, 15). However, we do not currently understand how the activity of HELB affects RPA filaments and vice versa.

**Fig. 1.**
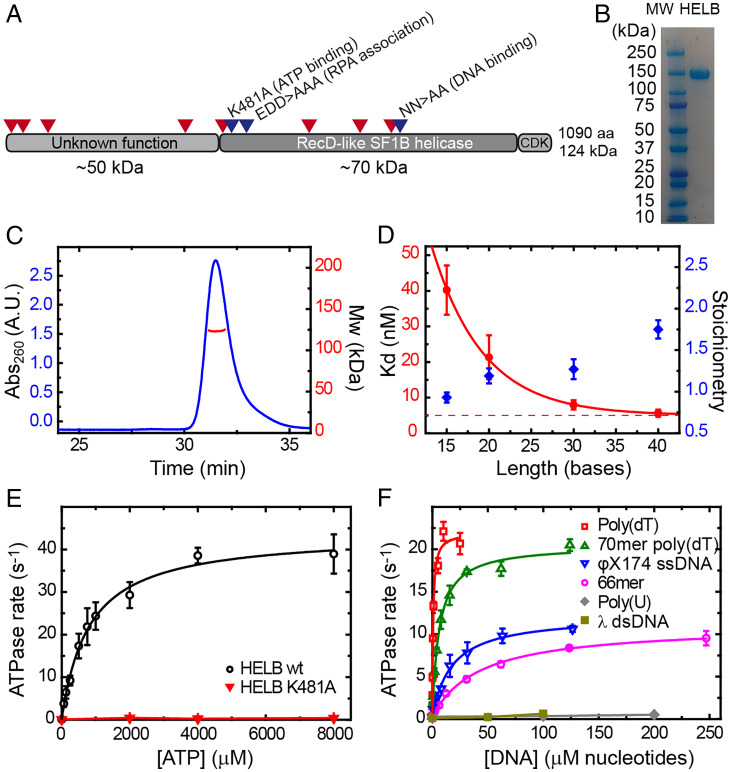
HELB is a monomer that binds tightly to ssDNA and displays ssDNA-dependent ATPase activity. (*A*) Cartoon of HELB showing overall domain layout and important mutations. Red marks denote positions of high-frequency tumor mutations. Blue marks denote positions of mutations that affect ATPase, DNA binding, and RPA binding activities. (*B*) Sodium dodecyl sulfate polyacrylamide gel electrophoresis (SDS-PAGE) analysis shows highly purified recombinant human HELB produced from insect cells. (*C*) SEC-MALS analysis demonstrates that HELB is a monomer in solution under these conditions with a calculated molecular mass (red data line) of 123,252 Da. (*D*, left axis) HELB binding constants (*K*_d_) for poly(dT) substrates of different lengths obtained in PIFE assays described in *SI Appendix*, Fig. S1*A*. An exponential fit determines a saturating *K*_d_ of 5 nM. (*D*, right axis) Stoichiometry values obtained under tight binding conditions as shown in *SI Appendix*, Fig. S1 *B*–*E*. (*E*) Michaelis–Menten plot of ATP hydrolysis gives *K*_m_ and *k*_cat_ parameters for WT HELB and also shows that the K481A mutant is unable to hydrolyze ATP. (*F*) Analysis of DNA stimulation of HELB ATPase activity demonstrates that HELB is an ssDNA-dependent helicase. ATP turnover is stimulated more by polythymidine substrates than mixed base sequences, likely due to their inability to form inhibitory secondary structures. A.U., arbitrary units; mw, molecular weight.

HELB is a 120-kDa protein comprising three distinct domains: an N-terminal region of unknown function, a central helicase domain sharing homology with the Superfamily 1 (SF1) helicase RecD, and a C-terminal region containing CDK phosphorylation sites (3) (Fig. 1*A*). Site-directed mutagenesis has implicated the central helicase domain in both the DNA and RPA binding activities of HELB (Fig. 1*A*, blue arrows). Interestingly, mutations in HELB are associated with both female infertility and early-onset menopause and are found widely distributed in the protein sequence in human tumor samples (Fig. 1*A*, red arrows) (16, 17). In vitro studies show that HELB possesses ssDNA-dependent ATPase activity and 5′ to 3′ helicase activity, which is as expected based on the similarity to RecD (2, 18). However, precisely how these biochemical properties underpin the cellular function(s) of HELB and the significance of the interaction with RPA are unresolved.

In this study, we used bulk and single-molecule assays to further characterize HELB, including its physical and functional interactions with RPA and RPA nucleoprotein filaments. Paradoxically, we find that human RPA (itself a potent ssDNA binding protein) is stimulatory to all activities of HELB on ssDNA, despite the competition one would expect between the two proteins for their nucleic acid substrates. In contrast, noncognate RPA protein inhibits all activities of human HELB. These highly specific interactions with RPA filaments help to recruit HELB onto ssDNA that is devoid of secondary structure and promote efficient ssDNA translocation coupled to the processive clearance of RPA. The implications of this finding for the roles of HELB in DNA replication and recombination are discussed.

## Results

### HELB Is a Monomeric Protein That Binds Tightly to ssDNA and Displays ssDNA-Dependent ATPase Activity.

Human HELB contains a C-terminal SF1 helicase domain and a large N-terminal region of unknown function, which displays no apparent homology to known proteins or folds (Fig. 1*A*). In order to better characterize this protein, we first prepared pure recombinant HELB from insect cells (Fig. 1*B*). Size exclusion chromatography combined with multiple angle light scattering (SEC-MALS) analysis showed that native HELB has a molecular mass of 123 ± 3 kDa, which is the expected value for a monomer (Fig. 1*C*). To quantitatively investigate ssDNA binding activity, we used a protein-induced fluorescence enhancement (PIFE) assay to measure binding to a series of poly(dT) oligonucleotides of increasing length (*SI Appendix*, Fig. S1*A*). This assay detects binding of protein within ∼3 nm of a dye label on one end of the DNA as an increase in fluorescence intensity. For a nonspecific interaction along the DNA lattice, the signal is maximal when the DNA is saturated. Under weak binding conditions (i.e., with [DNA] lower than *K*_d_), we observed that the relationship between fluorescence intensity and [HELB] was approximately hyperbolic, and the data were fit to yield *K*_d_ for each DNA length tested (*SI Appendix*, Fig. S1*A*). The affinity of HELB for ssDNA increased with increasing length until saturation at *K*_d_ ∼ 5 nM for oligonucleotides of 30 bases or longer (Fig. 1*D*), suggesting a binding site size of between 20 and 30 nucleotides. We then performed the PIFE assay under tight binding conditions (i.e., with [DNA] much higher than *K*_d_) to determine binding stoichiometry (*SI Appendix*, Fig. S1 *B*–*E*). We found that one HELB monomer binds to ssDNA molecules that are up to 30 bases in length, whereas two HELBs can be accommodated by a 40-mer oligonucleotide. Taken together, these data suggest a binding site size of ∼20 nucleotides, a conclusion that is further supported by electron mobility shift assays ( EMSA) (*SI Appendix*, Fig. S1*F*). This is larger than is typical for SF1 DNA helicases; structural studies of many representative examples of these enzymes show that their core helicase domains bind approximately eight nucleotides, and work with Rep helicase shows that two monomers can bind side by side to a 16-mer oligonucleotide (19, 20). This implies that, in addition to an expected ssDNA binding site in the core helicase domains, HELB contains an undefined DNA binding locus, perhaps in the N-terminal region of the protein, an idea that is consistent with further experiments presented below.

HELB displays ssDNA-dependent ATPase activity with Michaelis–Menten parameters of *k*_cat_ = 44 ± 3 s^−1^ and *K*_m (ATP)_ = 800 ± 140 µM measured in the presence of saturating poly(dT) concentrations (Fig. 1*E*). This turnover number is significantly higher than has been reported previously (280 ATP min^−1^) (2). As expected, substitution of the conserved lysine in the Walker A motif (helicase motif I) to alanine (K481A) dramatically reduced ATPase activity, showing that this activity is intrinsic to the purified HELB polypeptide (Fig. 1*E*). We compared the ability of six model nucleic acid substrates to stimulate the ATPase activity of HELB (Fig. 1*F*). Duplex DNA and poly(U) single-stranded RNA did not stimulate the ATPase rate. In contrast, ssDNA strongly activated ATPase activity. We found that poly(dT) (a mixture of polythymidine chains of average length of ∼1,000 nucleotides that is incapable of forming secondary structures) was a significantly better substrate for HELB than φX174 virion ssDNA (a circular 5,386-nucleotide molecule, which is expected to form extensive secondary structures), reflected in terms of both a higher *k*_cat_ and a lower *K*_DNA_. This indicates that secondary structure and/or DNA sequence may affect both the binding of HELB onto DNA and the DNA-stimulated ATPase that is coupled to translocation.

### HELB Translocates Efficiently on ssDNA in a 5′ to 3′ Direction.

**Fig. 2.**
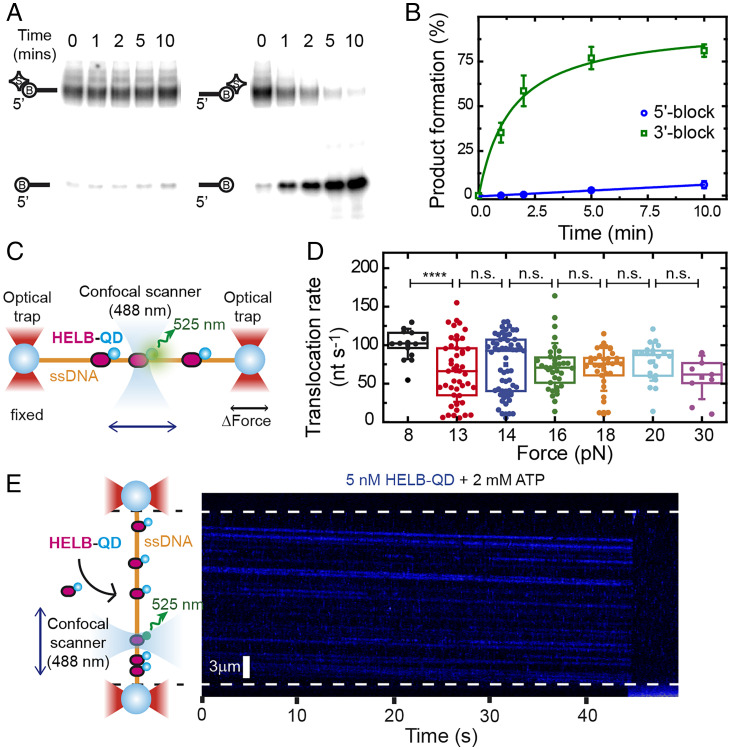
HELB efficiently translocates on ssDNA in a 5′ to 3′ direction. (*A*) The streptavidin displacement assay in bulk demonstrates that HELB moves along ssDNA specifically in a 5′ to 3′ direction. (*B*) Quantification of the gel-based assay. (*C*) Illustration of the experimental C-Trap^®^ setup. Individual λ-sized ssDNA tethers were attached between two streptavidin-coated beads trapped by two optical traps. A confocal laser scanned the DNA tethers to detect HELB conjugated to one QD. (*D*) Box plots of HELB translocation rates on ssDNA as a function of force in the presence of 2 mM ATP (9 ≤ *n* ≤ 59). Statistical analysis revealed no statistical significance of the mean rates at forces ranging from 13 to 30 pN (*P* > 0.05). The comparison of data at 8 pN with the rest of the forces gave 1.6·10^−6^ ≤ *P* ≤ 0.016, indicating that this population is significantly different. ****P < 0.0001. (*E*) A cartoon of a tethered ssDNA loaded with multiple HELB–QDs (*Left*) and a representative kymograph of HELB movement (blue) in the presence of 2 mM ATP under 18 pN of tension (*Right*).

To assess the putative ssDNA translocase activity of HELB, we first used an indirect gel-based assay based on the displacement of streptavidin from biotinylated oligonucleotides (21). By comparing streptavidin displacement from oligonucleotides labeled at either the 3′ or 5′ end with biotin, one can infer DNA translocation activity as well as its polarity. We observed that HELB efficiently removed streptavidin from 3′ biotin (but not 5′ biotin)–labeled substrates, suggesting that HELB moves in the 5′ to 3′ direction (Fig. 2 *A* and *B*). This is the expected polarity given the sequence similarity to the RecD family of helicases.

Next, to better characterize the rate and processivity of ssDNA translocation, we directly imaged the movement of fluorescent HELB on ssDNA using a combined optical tweezers and confocal fluorescence microscope (22, 23) (C-Trap; Lumicks). For these experiments, we first conjugated biotinylated HELB to streptavidin-coated quantum dots (QDs), a process that does not affect its ATPase activity (*SI Appendix*, Fig. S2*A*). A long ssDNA substrate was generated from a λ-phage double stranded (dsDNA) (48.5 kilo base pairs, kbp) that had been tethered between two optically trapped beads by applying a tension above the overstretching force in a low-salt buffer (Fig. 2*C* and *SI Appendix*, Fig. S2 *B* and *C*). Successful generation of an ssDNA molecule was assessed by its mechanical fingerprint, which is very different from dsDNA (*SI Appendix*, Fig. S2*D*). After a single ssDNA molecule was stably trapped between the beads, we moved the molecule into a channel containing 5 nM HELB–QD and 2 mM (saturating) ATP and recorded confocal images between the beads at 50 to 100 ms line^−1^, allowing us to build kymographs depicting HELB binding and movement on DNA (Fig. 2*E*). The DNA was maintained at a tension higher than 8 pN to avoid the formation of secondary structures on the ssDNA. These experiments showed that HELB binds to ssDNA and then translocates in the same direction on any given DNA molecule (Fig. 2*E* and *SI Appendix*, Fig. S2*E*). Because the ssDNA tether orientation is arbitrary, we cannot determine the translocation polarity. However, based upon our bulk experiments, movement is presumably in the 5′ to 3′ direction. In the absence of ATP, HELB was able to bind to ssDNA but remained stationary (*SI Appendix*, Fig. S2*F*). The rate of translocation on naked ssDNA at room temperature was found to be independent of the tension in the range of 13 to 30 pN (*P* value > 0.05) with a mean value of 72 ± 40 nt s^−1^ (peak ± width/2, *n* = 204) (Fig. 2*D*). The distribution of translocation rates at 8 pN was found to be statistically different from the others (1.6·10^−6^ ≤ *P* value ≤ 0.016) and encompasses larger values. Together, these experiments show that HELB is a processive motor protein that couples ATP hydrolysis to 5′ to 3′ unidirectional translocation along ssDNA without the need to initiate from a free DNA end.

### HELB Is an Efficient Helicase Only When Assisted by Force.

**Fig. 3.**
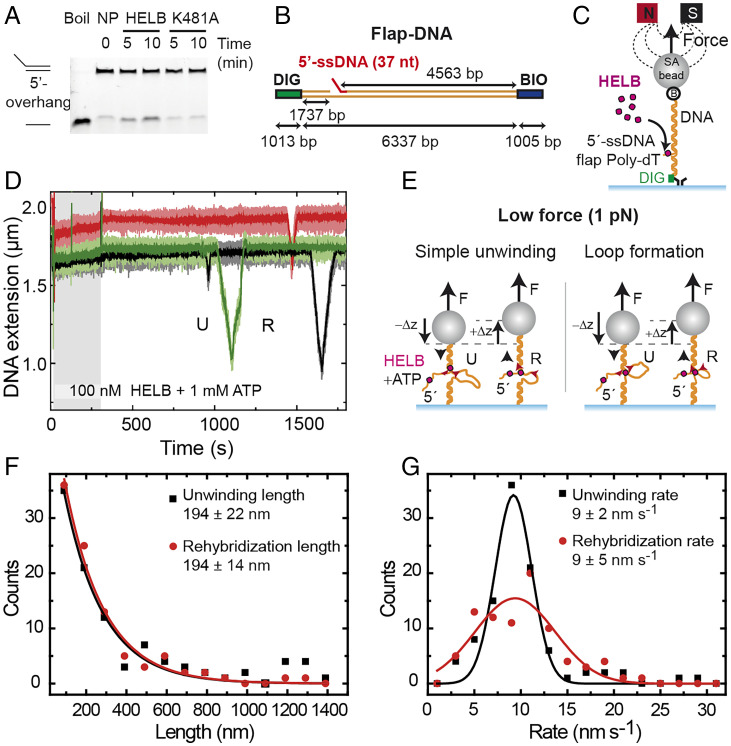
HELB can unwind duplex DNA and switch translocating strands. (*A*) A bulk unwinding (U) assay shows limited HELB duplex U activity. NP, no protein. (*B*) Schematic representation of the Flap-DNA substrate employed for MT single-molecule unwinding assays. Bio and Dig indicate DNA ends labeled with biotins and digoxigenins. (*C*) Cartoon of the MT assay for monitoring DNA unwinding by HELB. SA bead, streptavidin-coated bead. (*D*) Representative time courses of MT experiments performed at 1 pN with 100 nM HELB and 1 mM ATP. Several events where the DNA extension decreases (U) and then increases again (R) to reach the initial bead’s height are shown. (*E*) The U events can be explained by direct HELB U at low forces (exposing a stretch of ssDNA) or by loop formation. Upon strand switching, the HELB translocation along the opposite strand leads to the rehybridization (R) of the helix and the recovery of the initial height of the bead (R events). (*F*) Distribution of the U length (black) and R length (red) measured in MT time courses. Fittings to two exponential decays give the mean values of = 194 ± 22 nm (error of fitting, *n* = 99) and = 194 ± 14 nm (error of fitting, *n* = 94). (*G*) Distribution of the U rate (black) and R rate (red) of events extracted from MT time courses similar to those shown in *D*. Fits to Gaussian functions give the mean U rates of = 9 ± 2 nm s^−1^ (*n* = 99) and = 9 ± 5 nm s^−1^ (*n* = 89).

To assess the extent to which HELB couples its ssDNA translocation activity to helicase activity (i.e., strand unwinding and separation), we first performed bulk helicase assays using a short duplex DNA containing a 5′ overhang as a loading site (Fig. 3*A*). At elevated protein concentrations (500 nM), the wild type, but not ATPase-dead mutant HELB, partially unwound the duplex, revealing intrinsic helicase activity as reported previously (2). However, the apparently weak helicase activity detected on this substrate contrasts with the potent translocase activity reported above. We next performed magnetic tweezers (MTs) experiments to investigate further the kinetics of duplex unwinding by HELB and the potential role of the force in this activity. We fabricated an ∼6.3-kbp DNA substrate containing a 5′-terminated poly(dT) ssDNA (37 nt) positioned 1.7 kbp from one end that acts as a loading site for HELB, named as Flap-DNA (Fig. 3*B*). One DNA end was attached to a glass surface of a fluid cell, and the other was attached to a superparamagnetic bead. External magnets located above the cell were then used to apply a controlled force to extend the DNA while the height of the bead was monitored (Fig. 3*C*). In this setup, it is possible to monitor DNA unwinding because ssDNA and double-stranded DNA display different extension for a given force (24). In the high–applied force regime (*F* ≥ 6 pN), ssDNA is longer than duplex DNA, and so, helicase activity leads to an increase in the Z position of the bead. Under low forces (*F* < 6 pN), ssDNA is shorter than duplex, and unwinding leads to a reduction in the height of the bead (25, 26).

MT unwinding experiments were first performed in the low–applied force regime (1 pN). Upon addition of 100 nM HELB and saturating ATP, we observed cycles of unwinding, reflected in a reduction of the bead height, and rehybridization events, in which the initial extension was recovered (Fig. 3*D*). A control gap substrate was also prepared containing a 63-nt gap and no flap, named as Gap-DNA (*SI Appendix*, Fig. S2*G*). No activity was observed in control experiments using the Gap-DNA substrate, suggesting that unwinding initiates from the free 5′-ssDNA tail (*SI Appendix*, Fig. S2*H*). To estimate the number of unwound base pairs and the velocity, we measured the change in extension in time and considered two models (Fig. 3*E*). The first is based on the formation of expanding ssDNA regions (i.e., simple unwinding). The second, which requires an additional DNA binding site and is included for reasons that will become more apparent below, is based on ssDNA loop formation. In the simple unwinding scenario, HELB would have unwound 1,240 ± 140 bp (error of the exponential fit, *n* = 99) at an unwinding rate of 56 ± 11 bp s^−1^ (peak ± half width, *n* = 99). In the ssDNA looping model, HELB would have unwound 865 ± 146 bp at 45 ± 10 bp s^−1^, a rate very similar to that observed in the ATPase and ssDNA translocase assays above. Note that a combination of both models is also possible if HELB unwinds a section of the duplex and then forms a loop. Regardless of the model, the rehybridization parameters were similar to those obtained in unwinding time courses (Fig. 3 *F* and *G*). Moreover, the similar unwinding and rehybridization rates, as well as the apparent symmetry of these events, support the idea that they reflect strand switching by a single HELB enzyme (24). We conclude that HELB can processively unwind DNA at low force. It is important to note, however, that these events were extremely infrequent, as they were typically observed several minutes after injection of HELB (*SI Appendix*, Fig. S3*A*). These rare events observed at the single-molecule level at low force are consistent with bulk DNA helicase assays (at zero force), which detect only a very limited 5′ to 3′ helicase activity (Fig. 3*A*). Further control experiments performed in the absence of ATP (*SI Appendix*, Fig. S3*B*), with the ATPase-dead mutant K481A (*SI Appendix*, Fig. S3*C*), and on nicked DNA (*SI Appendix*, Fig. S3*D*) did not show any helicase activity.

**Fig. 4.**
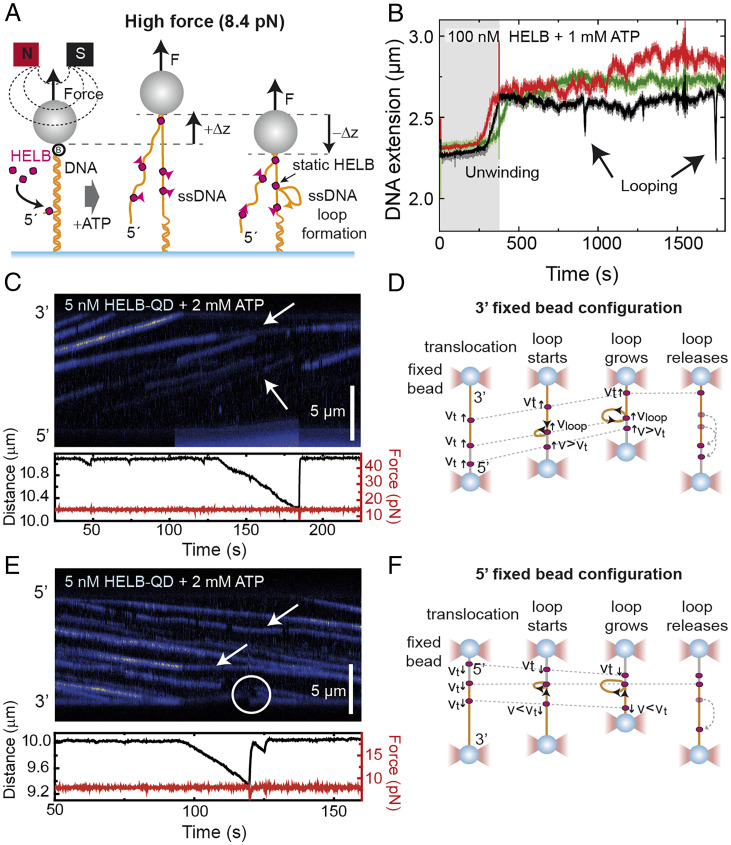
HELB efficiently unwinds duplex DNA when assisted by force and forms loops on ssDNA. (*A*) Schematic representation of a model for DNA unwinding and loop formation by HELB. The bead’s height increases at high force (i.e., *F* ≥ 6 pN) due to HELB unwinding of the duplex DNA by translocating from the 5′-ssDNA overhang. Exposure of a long ssDNA section will facilitate binding of additional HELB proteins, which will move with 5′ to 3′ polarity. A potential second binding site in HELB might facilitate the formation of a loop by keeping the protein still on the DNA. The formation of a loop results in a decrease of the bead’s height. Movement of HELB is indicated by pink arrows. (*B*) Representative time courses of MT experiments with 100 nM HELB and 1 mM ATP taken at 8.4 pN, showing characteristic unwinding and looping events. (*C*) Representative kymograph of HELB–QD (blue) translocating toward the immobile fixed bead in a force-clamp experiment (3′–fixed bead configuration). The formation of a loop is detected as a shortening of the extension of the tether (*Lower*). (*D*) Model to recapitulate the process of loop formation and release in the 3′–fixed bead configuration. As predicted, HELB particles downstream of the loop increase their apparent velocity (white arrows in *C*). (*E*) Representative kymograph of HELB–QD (blue) translocating from the immobile fixed bead in a force-clamp experiment (5′–fixed bead configuration). (*F*) Model to recapitulate the process of loop formation and release in the 5′–fixed bead configuration. As predicted, HELB particles at the loop appear immobile in the kymograph, and the release of the loop results in a sudden jump of HELB positions (white arrows and circle in *E*).

We next performed equivalent MTs experiments but at a higher applied force of 8.4 pN, conditions under which ssDNA is longer than duplex DNA (Fig. 4*A*). We typically observed a rapid elongation of the tethers consistent with HELB efficiently unwinding DNA (representative time courses are shown in Fig. 4*B*). The time required to observe any activity (activation time) for these events was exponentially distributed with a time constant of 147 ± 10 s (error of fitting, *n* = 44), much shorter than that observed in the low-force regime (*SI Appendix*, Fig. S3*E*). Moreover, this value corresponds to (and is, therefore, limited by) the arrival of the protein and ATP to the position of the tracked tethers in the middle of the fluid cell, which is governed by the low-flow velocity we use to avoid perturbations in bead tracking. Helicase activity was observed in 48 of 74 traces (65%), while 35% showed no change in tether extension, which we attribute to the potential absence of the 5′ flap in the DNA substrates. Additional control experiments performed at high force in the absence of ATP, with the ATPase mutant, or with nicked DNA did not show any helicase activity (*SI Appendix*, Fig. S3 *F*–*H*). The observed unwinding length at 8.4 pN was 3,600 ± 900 bp (peak of the distribution ± width/2, *n* = 48) (*SI Appendix*, Fig. S4*A*), which implies that HELB can translocate and unwind the entire substrate. The unwinding rate distribution provided a mean value of 38 ± 4 bp s^−1^ (peak ± half width, *n* = 47) (*SI Appendix*, Fig. S4*B*), which is slightly lower than the ssDNA translocation rate obtained in C-Trap experiments (Fig. 2*D*) but similar to the ATPase rate measured in bulk (Fig. 1*E*). Overall, we interpret these traces as showing that HELB efficiently couples ATP hydrolysis to helicase activity that is initiated from the flap in the 5′ to 3′ direction when a high assisting force has been applied. Improved DNA unwinding with increasing force has been predicted as a general feature of other helicases (27).

### HELB Translocation Can Result in the Formation of DNA Loops.

Following the rapid and processive DNA unwinding, our high-force time courses presented a more complex behavior with continuous changes in extension with no obvious directionality (Fig. 4*B*). Occasionally, we noticed that, despite the high restraining force, the bead’s height dropped rapidly followed by a recovery of the extension (Fig. 4*B*, arrows). Events of this type occurred in about half of our long time courses. Because ssDNA is longer than duplex under these conditions (24), these events could be caused either by the conversion of ssDNA into dsDNA (i.e., annealing activity) or by the generation of a DNA loop (28). The first option would imply a strand switch of the unwinding HELB enzyme followed by the reannealing of the previously separated strands. However, this scenario can be discounted because we clearly observe the bead proceeding to below the original height of the tether (*SI Appendix*, Fig. S4*C*). We, therefore, favor the second explanation, whereby the activity of HELB leads to the formation of large DNA loops. Such behavior requires that the enzyme oligomerizes and/or that the monomer has more than one DNA binding locus (Fig. 4*A*). We observed two different kinds of height recovery following looping: a sudden jump in the bead’s height (in 18 of 40 events) or a gradual recovery of its initial position (in 22 of 40 events), including some pausing in discrete steps (*SI Appendix*, Fig. S4*D*). Both behaviors are consistent with the looping model depending on the nature of translocating blockage that causes the generation of the loop and how that is resolved. The length of the unwinding events that result in the formation of a loop (looping length) measured at 8.4 pN was 438 ± 60 bp (error of the exponential fit, *n* = 40) (*SI Appendix*, Fig. S4*E*), and the looping rate was 33 ± 14 bp s^−1^ (peak ± half width, *n* = 37) (*SI Appendix*, Fig. S4*F*), similar to the rate of unwinding measured in the same experimental conditions (*SI Appendix*, Fig. S4*B*).

To verify the formation of DNA loops, we next performed optical tweezers experiments in force-clamp mode and observed shortening of the apparent tether length. In this assay, one of the optical traps moves to keep the applied tension constant, affecting the apparent movement of HELB molecules depending on their orientation. HELB particles located downstream of a protein that generates a loop and moving toward the fixed bead would show an apparently faster velocity (Fig. 4 *C* and *D*). In the opposite case, HELB particles moving away from the fixed bead will pause at the loop or move with an apparently reduced velocity (Fig. 4 *E* and *F*).

### Interaction of HELB with RPA-Coated DNA Increases ATPase Activity and Favors ssDNA Loop Formation.

**Fig. 5.**
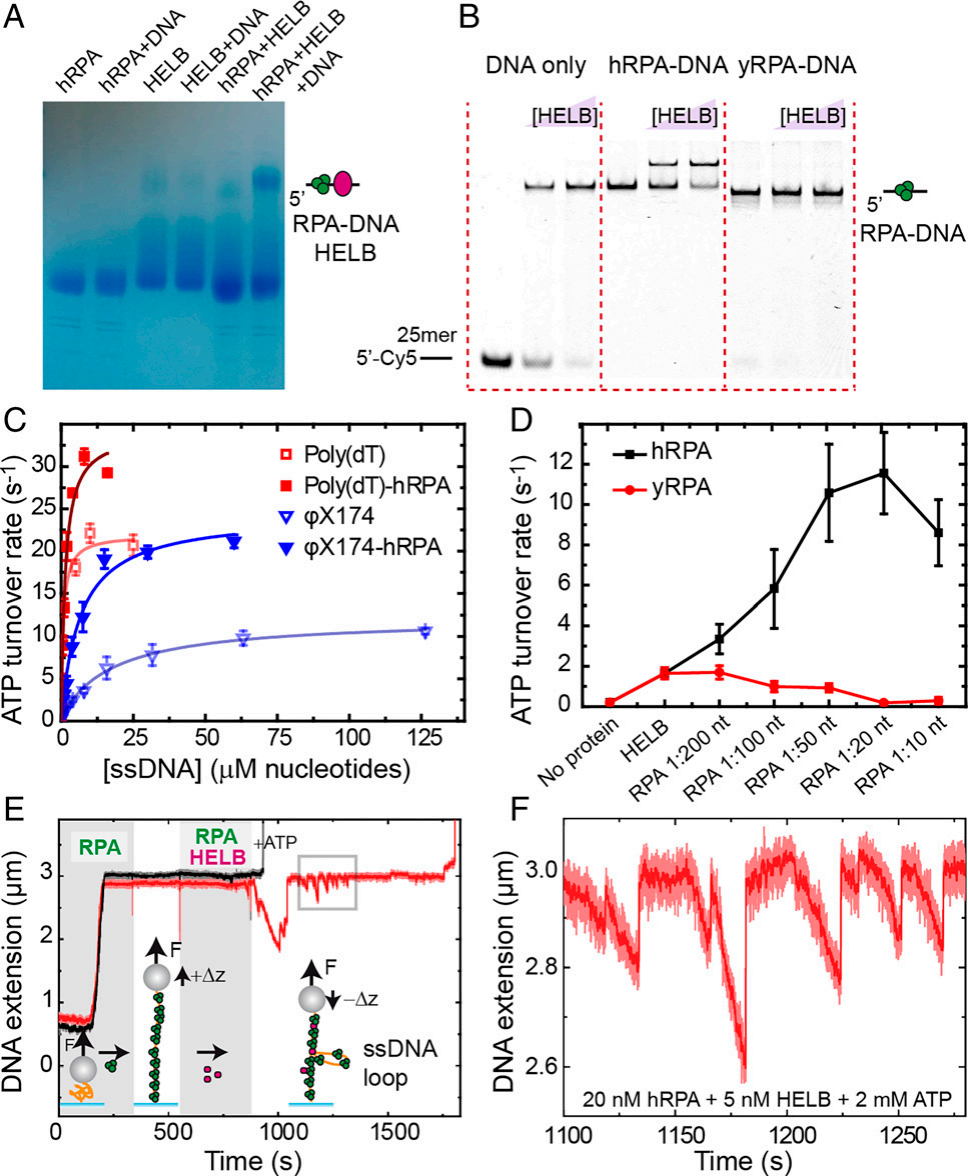
Interaction of HELB with RPA-coated DNA increases ATPase activity and favors ssDNA loop formation. (*A*) Native blue gel analysis confirms the formation of an HELB–human RPA–ssDNA complex with no apparent interaction observed for HELB and RPA without ssDNA. (*B*) Native PAGE EMSA analysis of HELB interaction with ssDNA, human RPA–coated ssDNA, and yeast RPA–coated ssDNA shows specific supershifted complex formation between HELB and human RPA–DNA. (*C*) The addition of RPA–ssDNA modifies HELB ATPase activity on the model substrates poly(dT) and virion φX174 (faded data are the same shown in Fig. 1*F*). In both cases, *k*_cat_ increases, but for the mixed base substrate, *K*_DNA_ also significantly decreases (*SI Appendix*, Table S3). (*D*) Stimulation of ATPase activity is unique to human RPA as yeast RPA causes inhibition of ATPase activity. (*E*) Example of an MT experiment where two ssDNA tethers covered with human RPA are measured under the effect of 5 nM HELB and 2 mM ATP (*F* = 3.8 pN). Shadow time windows indicate the flow of RPA and RPA + HELB + ATP. (*F*) Zoomed-in view of the square area marked in *E* to highlight the ssDNA looping dynamics promoted by HELB.

A direct interaction between HELB and RPA has been reported previously (4). However, using blue native polyacrylamide gel electrophoresis (PAGE) (Fig. 5*A*), we were only able to detect a complex between RPA and HELB in the presence of ssDNA. EMSA experiments using a 5′ Cy5–labeled 25-mer oligonucleotide demonstrated that HELB was able to supershift the RPA–DNA complex (Fig. 5*B*). Although this oligonucleotide is too short to fully accommodate both proteins side by side (RPA binds 20 to 30 nt, HELB binds ∼20 nt), we cannot exclude the idea that the apparent protein–protein interaction we see here is mediated by the DNA. Importantly, however, the ternary complex formed was species specific as no supershift was evident when substituting human RPA for *Saccharomyces cerevisiae* RPA (yRPA) (Fig. 5*B*).

We next investigated the effect of RPA on the ATPase parameters of HELB using two model ssDNA substrates: poly(dT) and φX174 virion (Fig. 5*C* and *SI Appendix*, Table S3). As shown above (Fig. 1*F*), in the absence of RPA, virion φX174 is a much poorer substrate for HELB [lower *k*_cat_ and dramatically higher *K*_DNA_ compared with poly(dT)]. We interpreted this as reflecting the high secondary structure content in virion DNA causing a physical block to binding and translocation. Naively, one might expect RPA (a tight ssDNA binding protein) to inhibit the ssDNA-dependent ATPase activity of HELB due to simple competition. However, we observed the opposite; substituting naked DNA for prebound DNA–RPA complexes (initially at one RPA:200 nucleotides, well below saturation) resulted in an increase in *k*_cat_ for both substrates. This implies that the presence of RPA on DNA is somehow stimulating the ATPase (and possibly, the translocation activity) of HELB in a manner unrelated to secondary structure content and potentially due to the direct physical interaction. Strikingly and selectively for the virion DNA, the addition of RPA also resulted in a marked decrease in *K*_DNA_ (i.e., apparently tighter binding). The fact that this did not occur with poly(dT) implies that RPA is facilitating recruitment of HELB to DNA by resolving DNA secondary structures that are otherwise unfavorable for binding.

To investigate whether stimulation of HELB ATPase activity by RPA was dose dependent, we performed an RPA titration experiment at a low concentration of φX174 ssDNA (0.1 × *K*_DNA_), where the observed ATPase rate is highly sensitive to stimulation (Fig. 5*D*). As RPA concentration increases from one RPA per 200 nt to one RPA per 20 nt (at which point we expect RPA to saturate the ssDNA), the observed rate of ATP hydrolysis increases by almost eightfold. At concentrations above saturation (one per 10 nt), there is a modest decrease from the maximum, suggesting either that free RPA in solution is somehow inhibitory to ATP hydrolysis in HELB or that recruitment of HELB to ssDNA may require short regions of naked ssDNA, such that RPA stimulates until close to saturation but inhibits as the nucleic acid lattice becomes filled completely. In complete contrast to the situation with human RPA, equivalent titrations with yeast RPA strongly inhibit HELB ATP turnover, presumably due to a simple competition for their substrates. This dose-dependent effect of RPA concentration and its species selectivity on HELB activity was also observed in bulk translocation assays (*SI Appendix*, Fig. S5 *A* and *B*). Taken together, these experiments show that the cognate RPA specifically recruits and stimulates the ATPase activity of HELB on ssDNA, implying that RPA nucleoprotein filaments are the physiological substrate for HELB.

Next, we investigated the interaction of HELB with RPA filaments using single-molecule techniques. We first prepared ssDNA tethers for MT experiments following the methodology described in ref. 26. Briefly, two strands of a dsDNA substrate are heat denatured followed by rapid cooling to avoid rehybridization. We employed a torsionally constrained construct based on the same insert that was used to fabricate the Flap-DNA substrate. Before proceeding with measurements, force–extension curves of the tethers ensured they were completely single stranded. The mechanical response of ssDNA with and without human RPA (hRPA) revealed an increase in extension, which was maximal at ∼3.8 pN (*SI Appendix*, Fig. S5 *C* and *D*). Further experiments confirmed that 20 nM RPA was saturating (*SI Appendix*, Fig. S5 *E* and *F*). At this applied force, the binding of 20 nM RPA produced an extraordinary increase of extension of the ssDNA tethers of approximately fourfold (Fig. 5*E*). Next, while maintaining the RPA concentration, we introduced 5 nM HELB and 2 mM ATP into the fluid cell and recorded the effect of HELB activity. In half of the time courses under these conditions (7 of 15 time courses), the HELB and ATP caused loss of the beads (Fig. 5*E*, black trace), probably because the translocating motor can disrupt the biotin–streptavidin bond holding the DNA to the bead. In the other half of the traces, we observed repetitive decreases in the bead’s height followed by a sudden recovery of the initial position (Fig. 5 *E*, red trace, and *F* and *SI Appendix*, Fig. S6 *A*–*C*). The reduction of the extension could be caused by the removal of RPA by HELB, as bare ssDNA has a shorter extension than the RPA filament. However, we can discard this possibility because free RPA is always present during the observed activity and would rebind the substrate on a faster timescale than HELB translocation, as clearly shown by the rapid extension rates observed upon initial RPA binding to ssDNA (Fig. 5*E*). Instead, we interpret the data in terms of a looping model, in which the HELB remains fixed to one strand while reeling in downstream ssDNA via the motor domains (which may or may not be coupled to the clearance of RPA). The sudden recovery in height is simply explained as dissociation of HELB from DNA and release of the loop. In complete contrast to these experiments with human RPA, no activity was observed in equivalent ssDNA MT experiments in the presence of 20, 100, and 500 nM yeast RPA (*SI Appendix*, Fig. S6 *D*–*F*). Together, these MT data suggest the possibility that HELB possesses a secondary static DNA binding site that facilitates the production of loops on RPA-coated ssDNA and further confirms the species specificity of the HELB–RPA interaction.

Intriguingly, bulk unwinding assays revealed a clear inhibitory effect on HELB helicase activity by RPA (*SI Appendix*, Fig. S7*A*). This was confirmed using MT-based unwinding assays, where we found that RPA always inhibited DNA helicase activity in Flap-DNA regardless of the applied force and whether the RPA was human or yeast in origin (*SI Appendix*, Fig. S7 *B* and *C*). A simple explanation could be that RPA prevents HELB loading to the free 5′ tail, but in any case, these results again focus our attention on RPA-coated ssDNA as the physiological substrate for HELB and question the relevance of any DNA unwinding activity in its cellular function.

### Direct Observation of HELB Translocation on ssDNA Shows That It Is Facilitated by and Causes Displacement of Human RPA.

**Fig. 6.**
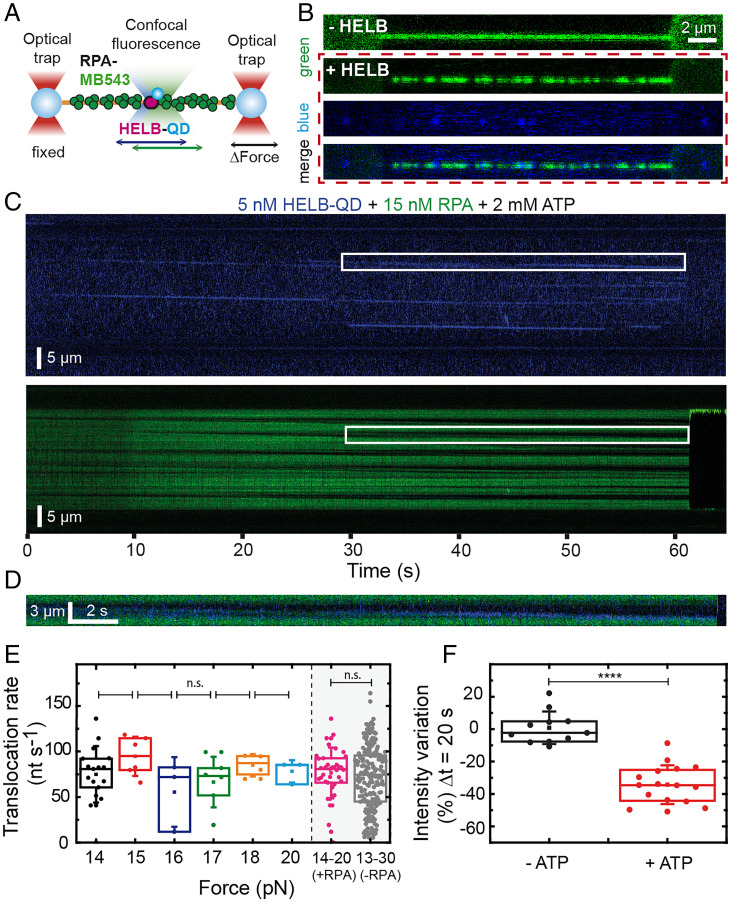
HELB translocation on ssDNA is facilitated by and causes displacement of human RPA. (*A*) Illustration of the experimental C-Trap setup. Individual ssDNA tethers attached to two optically trapped beads are covered with fluorescent hRPA^MB543^ and exposed to HELB–QD. Both proteins were detected by two-color excitation confocal microscopy. (*B*) 2D scans of a tethered ssDNA covered by hRPA^MB534^ in the absence of HELB (row 1) and after exposed to 5 nM HELB–QDs and 2 mM ATP filtered by green (RPA detection), blue (QDs detection), and merge fluorescence emission images. (*C*) Representative kymograph of HELB movement (blue) on ssDNA covered by hRPA^MB534^ (green) in the presence of 2 mM ATP. Signals obtained with blue and green emission filters are displayed separately. (*D*) Zoomed-in area of the regions marked in white in *C*, which contains the merged fluorescence of the blue and green channels. (*E*) Box plots of HELB translocation rates on hRPA-covered ssDNA in the presence of 2 mM ATP. *P* values > 0.05 indicate there are no significant differences between the populations shown. The distributions of data within the whole range of forces for hRPA–ssDNA (14 to 20 pN) and for bare ssDNA (13 to 30 pN) are included for comparison. (*F*) Variation within the 20-s time interval of the total fluorescence intensity measured in a 2-µm × 0.5-s time window. Control experiments without ATP and evaluated at similar time intervals did not show a significant decrease of intensity. ****P < 0.0001.

We next sought to directly characterize how HELB interacts with and affects RPA filaments using combined optical trapping with confocal scanning microscopy. We labeled human RPA with the MB543 (RPA^MB543^) fluorophore (emission 570 nm) and used the dual-color imaging ability of our instrument to simultaneously detect HELB–QD (blue channel) and RPA^MB543^ (green channel) (Fig. 6*A*). We first produced an ssDNA tether from λ-DNA as before. Then, we moved to a channel with 15 nM RPA^MB543^ and took a single image to confirm uniform coverage of the DNA by RPA (Fig. 6*B*, row 1). We next moved the nucleoprotein complex to a channel containing 5 nM HELB–QD and 2 mM ATP (and no RPA), and acquired video scans and kymographs between the two beads. Fig. 6*B* shows snapshots of a two-dimensional (2D) video scan (Movie S1), where the movement of HELB–QDs (represented as blue dots) on an RPA^MB543^–ssDNA filament (represented in green) is observed. Fig. 6*C* shows representative kymographs of the blue and green light excitation channels. We observed HELB trajectories in the blue channel moving unidirectionally on any given ssDNA molecule, as observed previously on bare ssDNA (Fig. 2*E*). The effect of HELB translocation on RPA distribution along the single strand of DNA was detected in the green channel. Interestingly, this was manifested as the progressive and unidirectional expansion of dark regions, which were apparently cleared of RPA. The overlap of the blue and green emissions confirmed that the HELB trajectories match with the progression of the dark front (Fig. 6*D* and *SI Appendix*, Fig. S8*A*). Notice, however, that in some dark regions of the RPA distribution, we did not detect any correlated QD emission signal (for example, the red arrows in the merged kymograph of *SI Appendix*, Fig. S8*A*). We attribute this to the presence of untagged HELB proteins that could not be detected. As expected, in the absence of ATP, we observed the binding of RPA and HELB, but the fluorescence signals remained static (*SI Appendix*, Fig. S8*B*). The translocation rate of HELB on RPA-covered ssDNA did not depend on the force in the range of tension applied (14 to 20 pN) (Fig. 6*E*). The mean rate deduced from the HELB–QDs trajectories (blue) was 82 ± 19 nt s^−1^ (peak ± half width, *R*^2^ = 0.92, *n* = 39) (Fig. 6*E*), similar to that on bare ssDNA (*P* value = 0.362 > 0.05). Analysis of the human RPA distribution intensity profiles as a function of time suggests that HELB translocation removes it from DNA. We compared the total intensity of a 2-µm × 0.5-s time window at the initial and final time (*t* = 20 s) of multiple translocation events. A large decrease of intensity compatible with the removal of RPA was observed in the presence of ATP. Photobleaching did not significantly contribute to this effect because control experiments without ATP showed no intensity decrease (Fig. 6*F*). Additional force-clamp experiments confirmed removal of RPA by HELB and clearly showed multiple HELB looping events, which resulted in the condensation of the DNA tether (*SI Appendix*, Fig. S9).

Equivalent experiments using fluorescent yeast RPA (yRPA^CY3^) gave strikingly different results. We observed that HELB binds poorly to ssDNA molecules covered by yRPA, and the reduced numbers of HELB proteins bound exhibit very limited movement (*SI Appendix*, Fig. S8*C*). Accordingly, the translocation rate distribution displayed a main peak around zero (*SI Appendix*, Fig. S8*D*). As we showed no interaction between yRPA and HELB (Fig. 5*B*), it is likely that the binding of the protein occurs in naked ssDNA regions, but HELB is blocked by yRPA and remains still. However, during the acquisition of long kymographs, we could detect movement of HELB, coupled with the clearance of yRPA from ssDNA. In this case, HELB moved at a similar rate to that observed in the presence of hRPA (*SI Appendix*, Figs. S6*E* and S8*D*) (*P* value 0.47 > 0.05; not significant [n.s.]). Together, these results are consistent with our previous findings showing that yeast RPA is inhibitory of the translocase activity of HELB.

## Discussion

In this work, we purified and characterized the human HELB helicase. This study confirms that the purified protein alone is an efficient ATPase and 5′ to 3′ ssDNA translocase. These properties are as expected based upon the similarity of the HELB helicase domain to the RecD-like family of SF1B helicases (19). The ATPase rates measured in bulk were broadly similar to the ssDNA translocation rates measured using single-molecule methods. Given the radically different nature of the assays used and the wide variability in individual translocation rates, it is likely that HELB hydrolyses one ATP for movement along each base of ssDNA as observed in studies of related helicases (29). We also demonstrated that ssDNA translocation is accompanied by the formation of DNA loops. Given that the protein is monomeric, this implies the presence of an additional static DNA binding domain beyond the translocating ssDNA binding site that is expected to be associated with the helicase core. The large binding site size observed here (∼20 bases) is also consistent with an additional unknown binding locus because SF1 helicase domains bind a stretch of only about eight bases (19). DNA looping is an emerging feature of processive helicases, which might help to suppress reannealing and therefore, improve duplex unwinding. However, HELB is in fact a very poor DNA helicase in vitro, and a cryptic ability to separate duplexes efficiently is only revealed by the application of an assisting force.

HELB was already shown to interact with the heterotrimeric RPA protein, which plays a central and ubiquitous role in DNA replication and repair in eukaryotic cells (9, 14, 30). Because of its high abundance and affinity for ssDNA, RPA was originally thought of as a protective factor for ssDNA. However, it is now appreciated that RPA nucleoprotein filaments can act as a dynamic platform for the recruitment or exclusion of other DNA binding proteins (31) or for initiating cell signaling cues (11). Moreover, the ability of RPA to “melt out” secondary or alternative structures in DNA, such as G quadruplexes, can also assist downstream processing of ssDNA intermediates: for example, the formation of uniform RAD51 filaments to promote DNA strand exchange. Nevertheless, these useful roles of RPA in managing ssDNA intermediates present a paradox. How can a very tightly bound ssDNA be handed off to additional enzymes that are required to complete replication or repair pathways? Interestingly, the RPA heterotrimer comprises six DNA binding domains joined by flexible linkers, and this modular organization potentially allows for other proteins to bypass or access ssDNA within RPA filaments, despite the very high-affinity interaction (14, 31). Such transactions may also be regulated by posttranslational modifications to RPA, the use of alternative RPA subunits, or the targeted remodeling of RPA filaments by additional factors (32). Interestingly, RPA filaments are known to interact physically and functionally with several helicase or translocase enzymes (15). These interactions, typically with a basic patch in the RPA70 subunit, help recruit and activate the motor proteins to their physiological site of action but may also have consequences for the formation, remodeling, or removal of the RPA filament (33). Among the best-studied examples are SF2 helicases, such as WRN and HelQ, whose DNA unwinding activity is stimulated by the cognate RPA protein (34, 35). Similarly, the helicase activity of the archaeal nucleotide excision repair and transcription factor XPD is facilitated by RPA2 (36). Remarkably, XPD can bypass RPA without displacing it, thereby overcoming its potential inhibitory effect as a roadblock to translocation (37). In contrast, bacterial SSB protein has been shown to be pushed along DNA by a variety of helicases without the need for any physical interaction, a phenomenon that may involve rolling the tetrameric SSB protein forward to avoid the need to completely disrupt its interactions with ssDNA (38).

Our work shows that the functional consequences of the HELB–RPA interaction are profound for both proteins but also quite distinctive compared with these other helicase systems. All ssDNA-dependent activities of HELB are stimulated by the presence of RPA. This phenomenon absolutely requires the cognate human RPA, and therefore, we presume a sustained physical interaction because the yeast ortholog inhibits all activities of HELB under the same conditions. This latter observation makes good sense because ssDNA binding proteins interact tightly with nucleic acids and must, therefore, compete with HELB for access to ssDNA. Quantitative analysis of the ssDNA-dependent ATPase activity of HELB in the presence and absence of RPA provides support for the idea that RPA stimulates HELB both as the result of a recruitment/loading phenomenon and by the activation of forward translocation, possibly because RPA premelts secondary structures that would otherwise slow the movement of the motor protein. Although it is both recruited and activated by its cognate RPA protein, HELB translocation activity then acts to remove RPA, leaving naked ssDNA in its wake, and duplex unwinding is in fact inhibited by RPA. We propose that the physiological target of HELB is the RPA–ssDNA complex and that HELB is an RPA displacement motor. This core biochemical activity, which does not preclude other functions for HELB, could potentially promote many DNA transactions where RPA filaments are intermediates that might otherwise block downstream processing events. In this respect, HELB may share certain aspects of its function with the yeast Srs2 helicase (13, 39). These findings have important implications for better understanding the biochemical basis for the roles that HELB might play in DNA repair and replication.

Although the in vivo function of HELB remains rather poorly defined, one may speculate on how an RPA clearance activity would be relevant to its proposed cellular roles (3). HELB localizes to replication origins and appears to play a role in the onset of chromosomal replication via interactions with both CDC45, a component of the replicative helicase, and DNA polymerase α-primase, which synthesizes RNA primers (2). Successful firing of replication forks involves association of RPA with ssDNA emerging from the CMG (Cdc45-MCM-GINS) helicases as they undergo activation at S phase, but RPA is also inhibitory to DNA polymerase α-primase (2, 40). Therefore, the RPA clearance activity of HELB observed here might facilitate the priming of DNA replication in the presence of the ssDNA binding protein. HELB also localizes to chromatin in an RPA-dependent fashion during replication stress and might, therefore, also act during replication elongation in the recovery of stalled forks (3, 4). In this context, RPA clearance might facilitate the origin-independent assembly of the replisome or repriming of leading strand replication. Finally, HELB has been proposed to both enhance (4) and inhibit (8) HR by facilitating strand exchange and inhibiting DSB resection, respectively. It is certainly easy to imagine how clearance of RPA in a 5′ to 3′ direction might promote strand exchange by facilitating the exchange of RPA for RAD51. Indeed, it is well established that the inherent affinity of RAD51 for DNA is insufficient to compete effectively with RPA in the absence of mediator proteins, such as BRCA2 (41, 42). In contrast, it is less immediately obvious how the 5′ to 3′ translocation polarity of HELB could directly inhibit the DSB resection nucleases, which generate 3′-ssDNA overhangs to initiate recombination. It is possible that removal of RPA might inhibit resection indirectly since RPA has been shown to enhance this early processing step in DSB repair (43). Alternatively, HELB might facilitate access to ssDNA for other factors that are inhibitory to DNA break resection and HR. Unpicking these possibilities will be the aim of future work.

## Materials and Methods

### Protein Expression and Purification.

A detailed description of human HELB, human RPA, and yeast RPA protein expression and purification (31, 44–46) is included in *SI Appendix*, *SI Methods*.

### Biochemical Assays for HELB Helicase.

The DNA binding activity of HELB was investigated using electrophoretic mobility shift and PIFE assays. ATP hydrolysis was monitored using a coupled pyruvate kinase/lactate dehydrogenase (PK/LDH) assay. ssDNA translocation was measured using streptavidin displacement from biotinylated oligonucleotides, and helicase activity was studied using strand displacement assays (21, 47, 48). Full details of these biochemical approaches can be found in *SI Appendix*, *SI Methods*.

### Blue Native PAGE.

Proteins and DNA were mixed in equimolar amounts (all 3 µM final concentration) in 20 mM Tris, pH 8.0, 200 mM NaCl, and 1 mM DTT. After 5 min of incubation at room temperature, samples were mixed with sample buffer, loaded onto a precast blue native gel, and run according to the manufacturer’s instructions (Life Technologies).

### Analytical SEC and SEC-MALS.

Proteins and DNA were mixed in equimolar amounts and loaded onto a Superose6 10/300 column equilibrated in 20 mM Tris, pH 8.0, 200 mM NaCl, and 1 mM TCEP. Chromatograms were recorded for the absorbance at 280 nm and at 260 nm against volume (milliliters) using Unicorn. SEC-MALS was used to determine the absolute molecular masses of full-length HELB. A 50-µg sample of HELB was loaded at 0.5 mL/min onto a Superose 6 10/300 size-exclusion chromatography (SEC) column (GE Healthcare) in 20 mM Tris, pH 8.0, 200 mM NaCl, and 1 mM TCEP using an Agilent High Performance Liquid Chromatography (HPLC). The eluate from the column was coupled to a DAWN HELEOS II MALS detector (Wyatt Technology) and an Optilab T-rEX differential refractometer (Wyatt Technology). ASTRA 6 software (Wyatt Technology) was used to collect and analyze light scattering and differential refractive index data according to the manufacturer’s instructions. Molecular mass and estimated error were calculated across individual eluted peaks.

### Optical Tweezers and Confocal Microscopy Assays.

Correlative tweezers–fluorescence experiments were performed at room temperature on an instrument combining three-color confocal fluorescence microscopy with dual-trap optical tweezers (C-Trap; Lumicks). A computer-controlled stage enabled the fast displacement of the optical traps within a five-channel fluid cell (*SI Appendix*, Fig. S2*B*). This microfluidic cell allowed for the in situ assembly and characterization of dumbbell DNA constructs and facilitated the direct transfer of the tethered DNA between different flow channels. Laminar flow-separated channels 1 to 3 were used to form a single-stranded biotin-λ DNA tether as follows; a single 4.38-µm streptavidin-coated polystyrene bead (Spherotech) was caught in each trap in channel 1 (trap stiffness of ∼0.4 pN/nm). The traps were then moved to channel 2 containing the biotinylated DNA intended for ssDNA fabrication by force. This DNA is a λ-phage dsDNA (Lumicks) biotinylated at the 3′ and 5′ positions of the same strand so that the nonbiotinylated strand can be removed by tension (49, 50). The traps were then moved to channel 3 containing a low–ionic strength buffer (10 mM Tris, pH 8.0, 1 mM EDTA) to favor the peeling. Here, the duplex DNA was held at forces higher than the overstretching transition, and the nonbiotinylated strand was removed (*SI Appendix*, Fig. S2*C*). The presence of an ssDNA was verified by force–extension curves (*SI Appendix*, Fig. S2*D*). Orthogonal channels 4 and 5 were used for protein loading and imaging.

To investigate the ability of HELB to bind and translocate on ssDNA, the tethers were moved into channel 5 containing 5 nM biotinylated HELB–QDs in 20 mM Tris, pH 7.5, 30 mM NaCl, 4 mM MgCl_2_, 5 mM DTT (reaction buffer), and 2 mM ATP. To test the behavior of HELB on an ssDNA covered by RPA, the single-stranded tethers were first moved to channel 4 containing 15 nM human RPA^MB543^ or yRPA^CY3^ in reaction buffer. The RPA-loaded tether was then dragged to channel 5 containing 5 nM HELB–QDs in the reaction buffer supplemented with 2 mM ATP. Flow was turned off during data acquisition. During force-clamp experiments, the force was kept constant at a target value using a feedback loop. The instrument is equipped with a multiwavelength laser engine for three-color confocal imaging. For our experiments, two excitation lasers were used: 488 nm for QDs and 525 and 532 nm for MB543 and Cy3 fluorophores. The emission was detected employing a blue filter 512/25 nm and a green filter 585/75 nm. Kymographs were generated via a confocal line scan through the center of the two beads.

Force and fluorescence data were analyzed using FIJI (51) and custom software provided by Lumicks. HELB velocity on bare ssDNA and in the presence of RPA was measured by dividing the distance traveled by the duration of each trajectory, Δ*L*/Δ*t* (*SI Appendix*, Fig. S8*A*). The HELB–QD trajectories were analyzed from the signal detected in the blue emission filter 512/25 nm.

### MT Assays.

We used an MT instrument setup similar to the one reported previously (52). Raw data were recorded at 120 Hz and filtered to 6 Hz for representation. Force values were calculated using the Brownian motion method applied to a DNA-tethered bead (53). Flap-DNA substrate (Fig. 3*B*) essentially consists of a DNA molecule of ∼6.3 kbp containing a flap poly(dT) tail of 37 nt in a specific site and flanked by two smaller fragments (∼1 kbp) that act as the immobilization handles as they are labeled with biotins or digoxigenins. The labeled parts are used to specifically bind each DNA end to a glass surface covered by antidigoxigenins and to streptavidin-coated magnetic beads. A control Gap-DNA substrate (with the gap but without a flap sequence) (*SI Appendix*, Fig. S2*G*) and a torsionally constrained dsDNA substrate (without a gap, employed to get ssDNA tethers) were also prepared. Doubly tethered beads were identified by applying magnet rotations on the beads and not considered for the analysis. DNA oligonucleotides used to fabricate MTs substrates can be found in *SI Appendix*, Table S1. The sequence of the DNA fragment used in this work can be found in *SI Appendix*, Table S4. Further details on the fabrication of MTs substrates and assays are included in *SI Appendix*, *SI Methods* (26, 28, 54–56).

### Statistical Analyses.

*P* values were determined from two-tailed two-sample *t* tests (n.s., *P* > 0.05; ****P < 0.0001). Box plots indicate the mean, median, and 25th and 75th percentiles of the distribution, and the whiskers show the SD.

## Supplementary Material

Supplementary File

Supplementary File

## Data Availability

All study data are included in the article and/or supporting information.
