# Supplementary material for: Human HELB is a processive motor protein that catalyzes RPA clearance from single-stranded DNA
Source: Proc Natl Acad Sci U S A. 2022 Apr 6;119(15):e2112376119. doi: 10.1073/pnas.2112376119 (PMC9169624; doi:10.1073/pnas.2112376119)
Supplement: Supplementary File [file pnas.2112376119.sapp.pdf]

PNAS
www.pnas.org

Supplementary Information for
Human HELB is a processive motor protein which catalyses RPA clearance from single-stranded DNA.

Silvia Hormeno[a,1], Oliver J. Wilkinson[b,1], Clara Aicart-Ramos[a], Sahiti Kuppa[c], Edwin Antony[c], Mark S. Dillingham[b,*], Fernando Moreno-Herrero[a,*]

[a] Department of Macromolecular Structures, Centro Nacional de Biotecnología, Consejo Superior de Investigaciones Científicas, 28049, Spain

[b] DNA:Protein Interactions Unit, School of Biochemistry, University of Bristol, BS8 1TD, UK

[c] Department of Biochemistry, Saint Louis University, MO 63104, USA.

[1] These authors contributed equally.

*Corresponding authors: Mark S. Dillingham, Fernando Moreno-Herrero.
Email: mark.dillingham@bristol.ac.uk , fernando.moreno@cnb.csic.es

This PDF file includes:

- Supplementary Methods
- Supplementary References
- Supplementary Figures S1 to S9
- Supplementary Tables S1 to S4
- Legend for Movie S1

Other supplementary materials for this manuscript include the following:

- Movie S1

Supplementary Methods

Human HELB protein expression and purification

A synthetic gene codon-optimised for *S. frugiperda* (Geneart, Invitrogen) encoding wild type human HELB was cloned into the pACEBac1 vector using the BamHI and XbaI restriction sites for use in the MultiBac system (Geneva Biotech). This was then screened for expression and purification using affinity tags in different positions. Following cleavage of a construct with a C-terminal 3C-cleavable StrepII tag, we obtained HELB in good yield and purity. The full-length recombinant protein as prepared (i.e. post-3C cleavage) contains a C-terminal extension -SGLEVLFQ (MW = 124,126 Da monomer), with the rest of the protein identical to UniProt entry Q8NG08. Mutagenesis (Quikchange XL, Agilent) was performed using this construct to create the K481A (ATPase) mutant. Bacmids were prepared by transposition of these plasmids and were used to transfect Sf9 cells in Insect Express media (Lonza) before viral amplification in the same cell line using standard techniques. For large scale expression, 500 mL of Hi5 cells at density 2 x 10^6/mL were infected with 25 mL of P3 virus and harvested by centrifugation after 70 hours at 27°C with shaking. The pellets were lysed into buffer containing 50 mM Tris pH 8.0, 100 mM NaCl, 1 mM DTT, 10% glycerol, protease inhibitor cocktail (Roche) and then sonicated on ice for a total of 2 minutes. After centrifugation at 4°C for 30 mins at 50000g, the cleared lysate was applied to Streptactin beads (GE Healthcare) in batch and incubated for 1 h at 4°C with rotation. After washing five times in batch with buffer containing 20 mM Tris HCl pH 8.0, 100 mM NaCl, 5% glycerol, 1 mM DTT, the protein was then eluted in the same buffer containing 2.5 mM desthiobiotin. The HELB-containing fractions were then applied to a 5 mL Heparin column (GE Healthcare). After washing, HELB was eluted with a gradient from 100 mM to 1 M NaCl over 16 CV in buffer containing 20 mM Tris HCl pH 8.0, 5% glycerol, 1 mM DTT. The HELB-containing fractions were pooled and digested for 3h at 4°C with 3C protease to remove the StrepII tag. The cleavage reaction was run over a 5 mL Streptactin column (Qiagen) to remove any uncleaved HELB and free StrepII peptide and the cleaved HELB-containing flow-through collected. This was spin concentrated (50,000 Da cutoff, Millipore) down to a volume of approximately 200 µL and applied to a Superose6 10/300 column in buffer containing 20 mM Tris HCl pH 8.0, 200 mM NaCl, 5% glycerol, 1 mM DTT. The HELB peak eluted after a volume of 15.5 mL, and was then concentrated using a centrifugal filter unit (Millipore) with glycerol added to a final concentration of 10% (v/v) and the protein stored at -80°C. Protein concentration was determined using a theoretical extinction coefficient of 120,780 M^{-1} cm^{-1}. Analysis of the purified protein by Orbitrap LC-MS/MS spectrometry was performed by the University of Bristol Mass Spectrometry Facility.

To produce biotinylated HELB, a modified version of the WT HELB pACEBac1 plasmid was constructed containing a C-terminal Avi-tag upstream of the cleavable StrepII tag. This protein expressed and purified in an identical manner to WT. After StrepII-tag cleavage and subsequent concentration to 50 µM, HELB-Avi was treated with BirA and biotin to site-specifically biotinylate the protein. After SEC purification (carried out as before) the protein was concentrated and then tested for biotinylation efficiency by PAGE gel (measured as ~100%). Conjugation of the biotinylated HELB protein to streptavidin did not affect its biochemical activities.

Human and yeast RPA protein expression and purification

Human RPA was produced using plasmid p11d-hRPA (a kind gift from Mark Wold, Univ. of Iowa) and purified from *E. coli* as described (Binz et al., 2006). Fluorescent *S. cerevisiae* RPA was purified and labeled with Cy3 as described previously using non-canonical amino acids (Pokhrel et al NAR 2017, Pokhrel et al NSMB 2019). Fluorescent human RPA labeled with MB543 was also generated using non-canonical amino acids (ncAA) as described for yeast RPA (1–3). Briefly, to obtain RPA carrying 4-aziodophenylalanine (4AZP), a p11d-hRPA-TAG32-107 plasmid was generated from p11d-hRPA. This plasmid contains a TAG at the chosen site of fluorophore incorporation, which corresponds to W107 in the human RPA32 subunit. A 6x-poly-histidine affinity tag was also engineered at the C-terminus of RPA32. This plasmid was cotransformed into BL21PlysS cells with pDule2-pCNF (4). This plasmid codes for the orthogonal $tRNA^{UAG}$ and tRNA synthetase for 4AZP incorporation. Cotransformants were selected using both ampicillin (100 µg/mL) and spectinomycin

(50 µg/mL). An overnight culture (10 mL) from a single colony was grown in LB media containing ampicillin and spectinomycin. 10 mL of the overnight culture was added to 1 L of minimal media. The minimal media for ncAA incorporation was prepared as previously described (4). Cells were grown at 37°C until the OD_{600} reached 2.0 and then induced with 0.4 mM IPTG along with 1 mM 4AZP. The 4AZP solution was prepared by first dissolving 206 mg in 250 µL of 5 M NaOH, vortexed extensively, and then adjusted to 8 ml with H_2O, and the entire mixture was added to 1 L of media to achieve a final concentration of 1 mM. Induction was carried out at 37°C for 3 hours. Harvested cells were resuspended in 120 mL cell resuspension buffer (30 mM HEPES, pH 7.8, 300 mM KCl, 0.02% Tween-20, 1.5X protease inhibitor cocktail, 1 mM PMSF, 10% (v/v) glycerol and 10 mM imidazole). Cells were lysed using 400 mg/mL lysozyme followed by sonication. Clarified lysates were fractionated on a Ni^{2+}-NTA agarose column. Protein was eluted using cell resuspension buffer containing 400 mM imidazole. Fractions containing RPA were pooled and diluted with H^0 buffer (30 mM HEPES, pH 7.8, 300 mM KCl, 0.02% Tween-20, 1.5X protease inhibitor cocktail, 10% (v/v) glycerol and 25 mM EDTA pH 8.0) to match the conductivity of buffer H^{100}, and further fractionated over a fast-flow Heparin column. RPA was eluted using a linear gradient H^{100}–H^{1500}, and fractions containing RPA were pooled and concentrated using an Amicon spin concentrator (30 kDa molecular weight cut-off). The concentrated RPA was next loaded onto a S200 column and fractionated using RPA storage buffer (30 mM HEPES, pH 7.8, 30 mM KCl, 0.25 mM EDTA, 0.02% Tween-20, and 10% (v/v) glycerol).

To fluorescently label RPA, RPA^{AZP} (~ 4 µM in 5 mL of storage buffer) was mixed with 1.5-fold molar excess DBCO-MB543 (Click Chemistry Tools Inc., AZ) or DBCO-Cy3. The reaction was incubated for 2 hours at 4°C and the labeled RPA was separated from free dye on a Biogel P4 column and resolved using RPA-storage buffer. An additional 10% glycerol was added to the fluorescent RPA protein and flash frozen using liquid nitrogen and stored at –80°C. RPA concentration was measured spectroscopically using e_{280} = 98500 $M^{-1}cm^{-1}$ (yRPA) or 87,210 $M^{-1}cm^{-1}$ (hRPA) and corrected for fluorophore contributions as described (1).

Electrophoretic Mobility Shift DNA binding assays

5′-Cy5-labelled DNA substrates (**Supplementary Table S2**) (2.5 nM final or 5 nM in **Figure S1F**) were mixed with increasing amounts of HELB protein in a total volume of 10 µL 1X EMSA buffer (20 mM Tris HCl pH 8.0, 100 mM NaCl, 1 mM DTT, 0.1 mg/mL BSA, 5% glycerol) and then incubated for 10 min at 25°C. In the case where RPA was used, 20 nM final concentration was added to the DNA before incubation with HELB. The samples were then loaded onto a 6% polyacrylamide (29:1) native 1xTBE gel and separated by electrophoresis in 1xTBE at 150V for 40 mins. The gels were visualised using a Typhoon scanner and analysed using ImageQuant software.

PIFE DNA binding assays

3′-Cy3-labelled DNA substrates (**Supplementary Table S2**) (2 nM final) were mixed in a quartz cuvette (Hellma) with increasing amounts of HELB protein in a total volume of 150 µL 1X PIFE buffer (20 mM Tris HCl pH 8.0, 100 mM NaCl, 1 mM DTT). After 1 min, the protein DNA mixture was scanned (λ_{Ex}=530 nm, λ_{Em}=562 nm, 5 nm slit widths) and the fluorescence intensity measured in arbitrary units. The fully saturated DNA-HELB gave an increase in fluorescence of around 60% compared to the original signal of free DNA. The measurements were normalised with the initial reading being 0 and the highest reading being 100%. For the purposes of determining the affinity and stoichiometry of HELB:ssDNA interaction it was assumed that each association of HELB monomers gave rise to the same fluorescence signal, allowing data to be fitted with standard equations for weak and tight binding regimes. Note that this is a simplification because the signal in the PIFE assay is also affected by position of binding. Nevertheless, we observed empirically that the data were well fit to these simple models. For K_d determination, data were fitted to a weak binding isotherm $y = B_{max} \cdot x/(x + K_d) + C$, where K_d is the binding constant, x the protein concentration, and B_{max} the substrate bound under saturating conditions. For the binding stoichiometry plots, DNA was used at high concentration (as stated in the plots) and the

experiments were carried out as before. These data were fitted to a version of the tight binding equation

$$y = B_{max}\left(\left(x + K_d + (S \cdot L)\right) - \sqrt{\left(x + K_d + (S \cdot L)\right)^2 - 4 \cdot x \cdot (S \cdot L)}\right)\Big/\left(2 \cdot (S \cdot L)\right) + C$$

, where S stands for stoichiometry (number of proteins bound to the DNA substrate), L is the DNA concentration, K_d is the binding constant, and x the protein concentration.

ATPase assays

ATPase activity was measured by coupling the hydrolysis of ATP to the oxidation of NADH which gives a change in absorbance at 340 nm. Reactions were performed in a buffer containing 20 mM Tris-HCl pH 8.0, 50 mM NaCl, 1 mM DTT, 5 mM $MgCl_2$, 50 U/mL lactate dehydrogenase, 50 U/mL pyruvate dehydrogenase, 1 mM PEP and 100 µg/mL NADH. Rates of ATP hydrolysis were measured over 1 min at 25°C. For calculation of K_{DNA}, the ATP concentration was fixed at 4 mM and the Michaelis-Menten plot was performed at [Poly(dT)] =10 x K_{DNA}. The concentration of HELB was 50 nM in these assays unless stated otherwise. In the cases where different DNA substrates and/or RPA were used, their concentrations are stated in the plots and/or figure legends.

ssDNA translocation assays

Streptavidin displacement assays were based on the method of Morris and Raney (5) with minor modifications as in (6). 5 nM (molecules) of 5′-^{32}P-labelled substrate 45mer oligonucleotides (**Supplementary Table S2**) were incubated with 400 nM streptavidin in 25 mM Tris-HCl pH 8, 50 mM NaCl, 4 mM $MgCl_2$, 1mM TCEP. Substrates were modified with either a 5′ or 3′ biotin moiety as indicated. The reaction was initiated by adding an equal volume of protein solution in the same buffer to give final concentrations of 250 nM HELB, 5 mM ATP and 8 µM biotin. The reaction was incubated at 37°C and stopped at certain points within a 10 min time course by quenching with an equal volume of stop buffer (300 mM EDTA, 400 mM NaCl, 30 µM poly(dT)). The products were separated on 10% polyacrylamide 1xTBE gels and visualised by phosphorimaging using a Typhoon imager. In the case where RPA was included, the reactions were set up as before but stopped after five minutes, and the final RPA concentrations used were 0, 25, 50, 100 and 200 nM.

Helicase (strand displacement) assays

Strand-displacement assays were based on a modification of the method of Matson (7). 10 nM (molecules) of 5′-Cy5-labelled oligonucleotides (**Supplementary Table S2**) consisting of a 25 bp duplex region with a flanking ssDNA overhang of 20 nt were incubated with 500 nM HELB in 20 mM Tris-HCl pH 8.0, 4 mM $MgCl_2$, 4 mM ATP, 1 mM DTT for 5 min at 25℃. The reaction was quenched by adding an equal volume of stop buffer (200 mM EDTA, 1% SDS, 10% (w/v) Ficoll 400 and 100 nM of an unlabelled form of the labelled strand in the substrate. In the cases where RPA was included, the final concentration was 100 nM. The products were separated on 15% polyacrylamide 1xTBE gels and visualised using a Typhoon imager.

DNA substrates for magnetic tweezers experiments

The Flap-DNA substrate for magnetic tweezers contains a 5′-ssDNA overhang or flap sequence of 37 poly(dT) nucleotides at a specific-site. It consists of a central fragment ligated to two digoxigenin or biotin-labelled DNA handles. The substrate is based on the pNLrep plasmid (8) that has a DNA sequence with five closely-spaced BbvCI restriction sites. Nicking of one of the two strands with Nt.BbvCI enzyme results in the formation of short 15–16 bases long fragments after heat denaturation, leaving a 63 nucleotides gap, where desired oligonucleotides can be hybridised.

The Flap-DNA substrate was fabricated following the protocol described in (9) with slight modifications. The pNLrep plasmid was digested with KpnI and PsiI enzymes (NEB) giving a 6337 bp product. To avoid reannealing of Nt.BbvCI cleavage products, a 100X excess of short

oligonucleotides complementary to the four released Nt.BbvCI-fragments (**Supplementary Table S1**) were added after Nt.BbvCI digestion and before inactivation of the enzyme for 20 min at 80°C. Following enzyme inactivation, the sample was cooled down to 40°C at a 1°C/1 min rate. The 6337 bp product with a 63 nt-gap was then purified with a PCR purification kit from QIAGEN and a 150X excess of Poly(dT)-flap oligonucleotide (**Supplementary Table S1**) was hybridised as described in (9). This generated a substrate as shown in (**Figure 3B**) with a duplex region of 4563 bp from the base of the Poly(dT)-tail oligonucleotide to the magnetic bead and a 37 nt poly(dT) tail. Handles were PCR-generated from the plasmid pSP73-JY0 (10) using appropriate oligonucleotides (**Supplementary Table S1**) and Bio-dUTP or Dig-dUTP (Roche), followed by restriction with PsiI or KpnI. The labelled fragments were ligated to the central part and the excess of oligonucleotides removed using two Microspin S-400 columns. DNAs were never exposed to intercalant dyes or UV radiation during their production and were stored at 4°C.

The Gap-DNA substrate was prepared as the Flap-DNA substrate but omitting the steps of hybridisation of the Poly(dT)-tail oligonucleotide after digestion with Nt.BbvCI.

A torsionally-constrained DNA substrate was prepared as the Flap-DNA substrate but omitting the steps of digestion with Nt.BbvCI to create the gap. A small fraction of these molecules were nevertheless nicked and were used in control experiments as Nicked DNA substrates.

To produce single-stranded DNA molecules for MT experiments we followed a protocol previously described in (11). Briefly, it consists in heating at 95°C for 5 minutes the stock solution of DNA in buffer TD (10 mM Tris-HCl pH 8, 20 mM DTT) followed by fast cooling to 4°C. For this purpose, we employed a torsionally-constrained DNA construct based on the same insert that was used to fabricate the DNA with a flap. In this case, the DNA was not treated with nucleases to generate any gap so that the molecule had no nick and was labelled with digoxigenins and biotins in both strands of the corresponding handles. The separation of the two strands by heat provided us with two single-stranded DNA molecules which can bind the surface and the bead in our assay. Note that we have the same probability of finding the biotinylated handle at 3′-end and at the 5′-end. The ssDNA molecules were then introduced into the fluid cell in ice-chilled TD buffer and incubated for several minutes. Then, we flowed in 2.8 µm paramagnetic beads covered by streptavidin (M280, Invitrogen) and incubated them briefly. After washing to remove the non-bound molecules and beads, we applied force with vertical alignment magnets with a 1 mm gap to locate the ssDNA tethers.

Magnetic tweezers unwinding assays

Single-molecule magnetic tweezers unwinding experiments were carried out at ambient temperature and at 1 or 8.4 pN as indicated, in a reaction buffer that contained 20 mM Tris-HCl pH 7.5, 30 mM NaCl, 4 mM $MgCl_2$, 5 mM DTT. To initiate the reaction, 100 nM HELB and 1 mM ATP were flowed into the fluid cell at 20 µL/min while the positions of the beads were measured by video microscopy. The injection of proteins is indicated as shadowed regions in the time courses. A fluid cell made with two parafilm layers (100 µL volume) and vertical alignment magnets with 0.2 mm gap were used to reach high applied forces using 1 µm beads (Dynabeads, Invitrogen). We chose to work at this concentration of HELB because we detected very limited activities below 100 nM, insufficient to manage a proper statistical analysis.

The quoted distances in base pairs were calculated from changes in DNA extension considering the different stretching properties of ssDNA and dsDNA. We experimentally determined the mechanical properties of dsDNA and ssDNA from force-extension curves in HELB's reaction buffer. For dsDNA we use the value given by the worm-like chain model of rise per base pair at a given force. For ssDNA, fits to the Freely Jointed Chain model at forces larger than 8 pN, where the formation of ssDNA secondary structure is prevented by force, resulted in a contour length per base of 0.66 nm nt^{-1}, in agreement with previously reported values (12).

Conjugation of HELB with quantum dots for optical tweezers experiments

To label HELB with QDs we incubated a 1:5 molar ratio solution of biotinylated HELB and streptavidin-coated QDs 525 (Q10143, Invitrogen) for 30 min on ice. We then added 1 mM biotin (B4501, Sigma) to neutralize the streptavidin molecules not bound to HELB. After 10 min the mixture was diluted in the reaction buffer to a final concentration of 5 nM HELB-QD and readily used.

Supplementary References

1. S. Kuppa, N. Pokhrel, E. Corless, S. Origanti, E. Antony, Generation of Fluorescent Versions of Saccharomyces cerevisiae RPA to Study the Conformational Dynamics of Its ssDNA-Binding Domains. *Methods Mol. Biol.* **2281**, 151–168 (2021).
2. N. Pokhrel, *et al.*, Monitoring Replication Protein A (RPA) dynamics in homologous recombination through site-specific incorporation of non-canonical amino acids. *Nucleic Acids Res.* **45**, 9413–9426 (2017).
3. N. Pokhrel, *et al.*, Dynamics and selective remodeling of the DNA-binding domains of RPA. *Nat. Struct. Mol. Biol.* **26**, 129–136 (2019).
4. J. T. Hammill, S. Miyake-Stoner, J. L. Hazen, J. C. Jackson, R. A. Mehl, Preparation of site-specifically labeled fluorinated proteins for 19F-NMR structural characterization. *Nat. Protoc.* **2**, 2601–2607 (2007).
5. P. D. Morris, K. D. Raney, DNA helicases displace streptavidin from biotin-labeled oligonucleotides. *Biochemistry* **38**, 5164–5171 (1999).
6. J. T. Yeeles, E. J. Gwynn, M. R. Webb, M. S. Dillingham, The AddAB helicase-nuclease catalyses rapid and processive DNA unwinding using a single Superfamily 1A motor domain. *Nucleic Acids Res* **39**, 2271–2285 (2011).
7. S. W. Matson, S. Tabor, C. C. Richardson, The gene 4 protein of bacteriophage T7. Characterization of helicase activity. *J. Biol. Chem.* **258**, 14017–14024 (1983).
8. N. Luzzietti, *et al.*, Efficient preparation of internally modified single-molecule constructs using nicking enzymes. *Nucleic Acids Res.* **39**, e15–e15 (2011).
9. O. J. Wilkinson, C. Carrasco, C. Aicart-Ramos, F. Moreno-Herrero, M. S. Dillingham, Bulk and single-molecule analysis of a bacterial DNA2-like helicase-nuclease reveals a single-stranded DNA looping motor. *Nucleic Acids Res.* **48**, 7991–8005 (2020).
10. N. Fili, *et al.*, Visualizing helicases unwinding DNA at the single molecule level. *Nucleic Acids Res* **38**, 4448–4457 (2010).
11. C. Carrasco, *et al.*, Dynamics of DNA nicking and unwinding by the RepC-PcrA complex. *Nucleic Acids Res.* **48**, 2013–2025 (2020).
12. A. Bosco, J. Camunas-Soler, F. Ritort, Elastic properties and secondary structure formation of single-stranded DNA at monovalent and divalent salt conditions. *Nucleic Acids Res* **42**, 2064–2074 (2014).

Supplementary Figures and legends

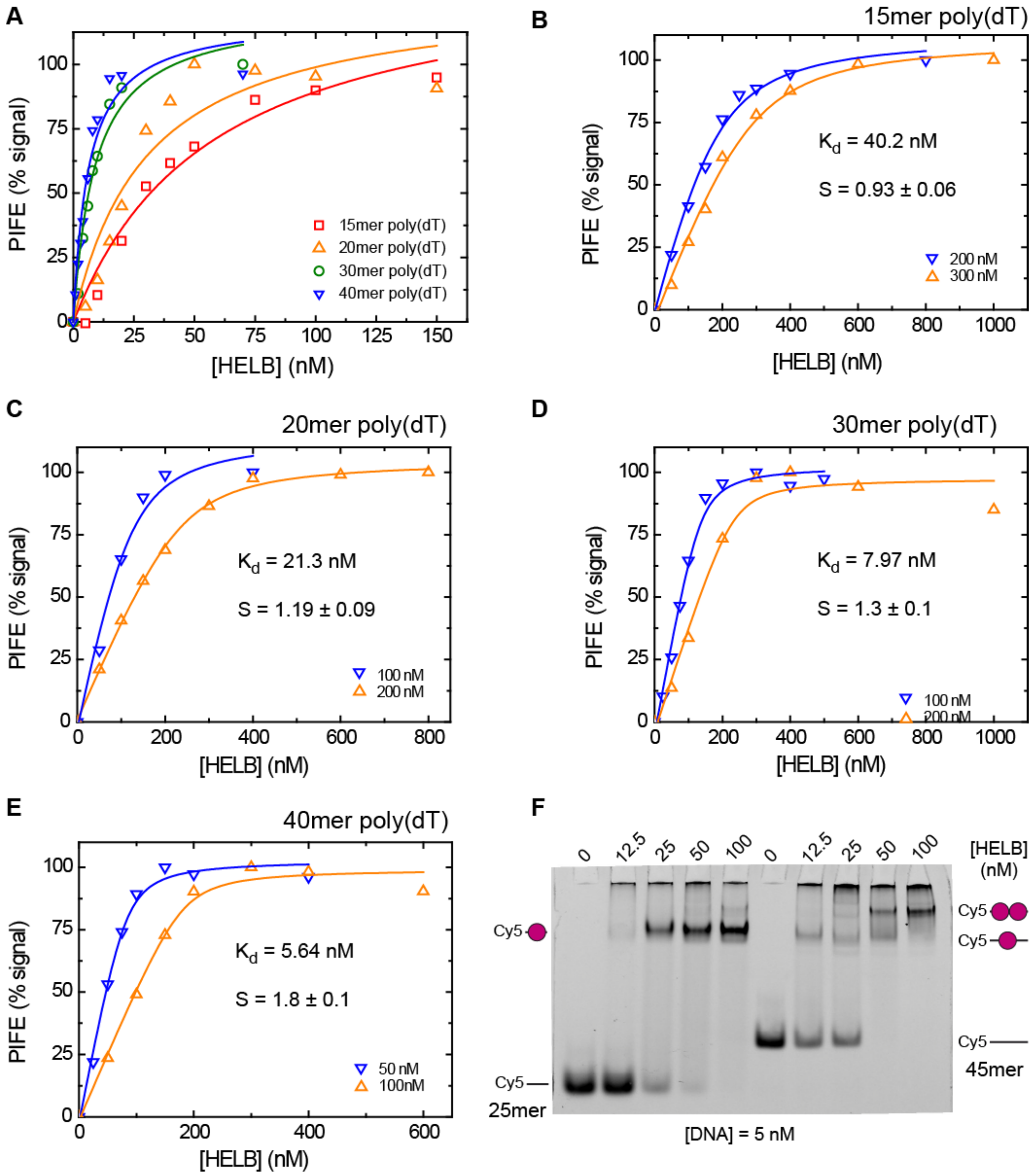

Fig. S1. *HELB binding constant and binding stoichiometry determination*

(A) PIFE DNA binding assay at low DNA concentration (<K_D) shows that tightest binding occurs with ssDNA of length >30 bases suggesting the presence of an additional DNA binding site in HELB other than that in the RecD-like domain. (B-E) PIFE DNA binding assays performed at high DNA concentrations (>>K_D) and fitted to the tight binding equation allow the binding stoichiometry to be determined for each length of DNA tested. Together, the data suggest a DNA-binding site size for HELB of approximately 20 nucleotides and is consistent with the presence of an additional DNA binding domain outside the RecD-like helicase domain. (F) Electrophoretic mobility shift assays were performed as described in the Methods. 5 nM Cy5-labelled ssDNA (either a 25mer or a 45mer as indicated) was incubated with the indicated concentration of HELB. Protein-DNA complexes and free DNA were then resolved by electrophoresis on a 6% native polyacrylamide gel. Note that the 25mer ssDNA predominantly forms a single shifted complex (which we interpret as a 1 HELB: 1 DNA), whereas the 45mer ssDNA forms two shifted complexes (which we interpret as 1 HELB: 1

DNA and 2HELB: 1 DNA). These results are therefore consistent with our interpretation of the PIFE assay DNA binding data, in which we conclude that HELB has an occluded site size of about 20 nucleotides.

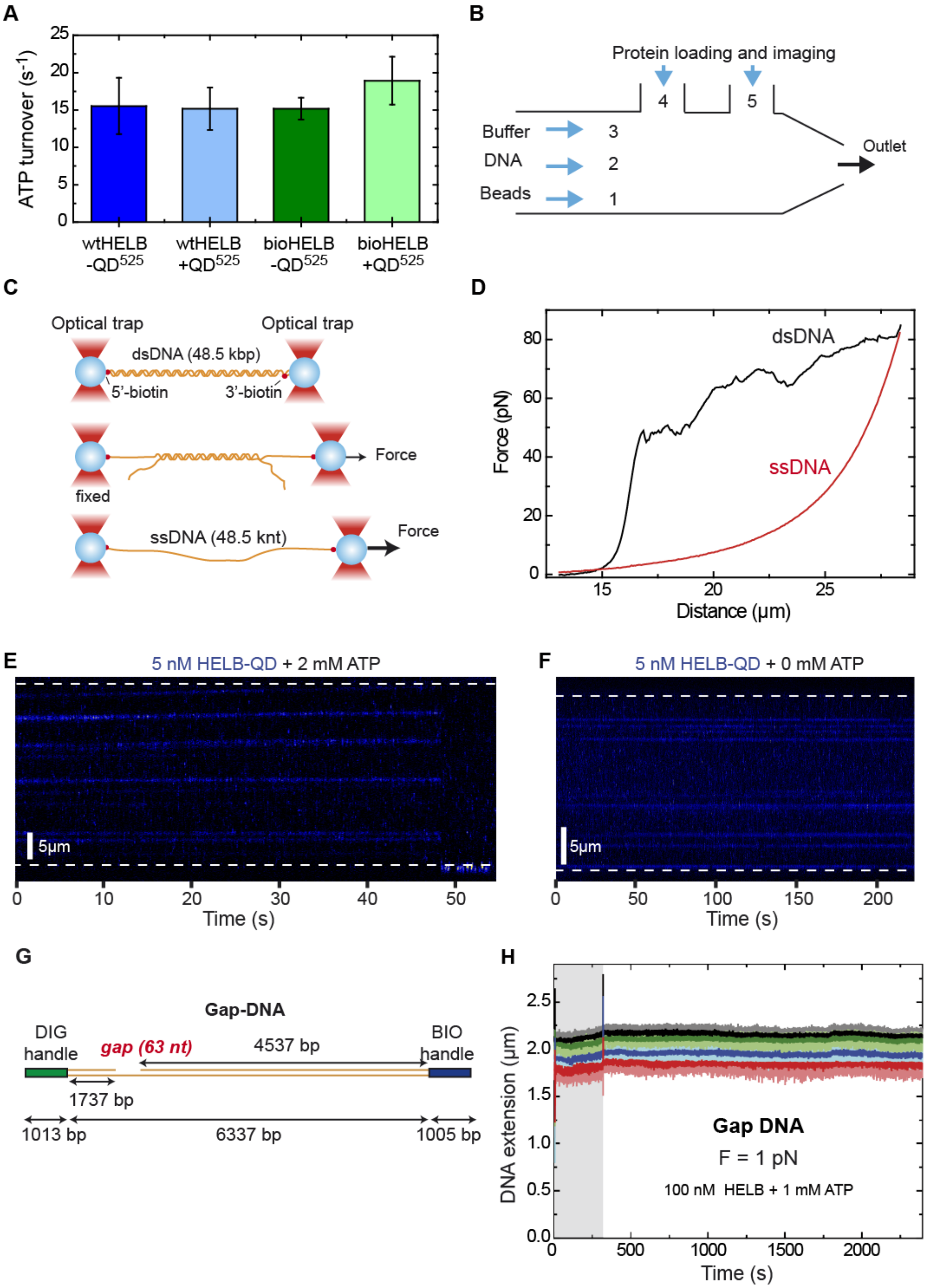

Fig. S2. *Control experiments and setup for optical tweezers experiments*

(A) ATPase assays were performed as described in the Methods with the following modifications. 100 nM wild type or biotinylated HELB was incubated with or without 500 nM Quantum dots (QD525, Invitrogen) for 10 minutes. ATPase reactions were initiated by adding 1/100th volume HELB-QD complexes (i.e. 1 nM final HELB) to a buffer containing 4 mM ATP, 7 μM poly(dT) as

well as other components of the coupling assay. Biotinylation and conjugation to QDs does not affect the ATPase activity of HELB. (B) Schematic of the experimental fluid cell used for optical tweezers experiments. Individual DNA tethers were formed in channels 1-3 separated by laminar flow containing streptavidin-coated beads, biotinylated λ-DNA for single-stranded DNA formation and a buffer with low salt, respectively. After ssDNA formation, the traps were subsequently moved to channels 4 and 5 for protein loading and imaging. (C) Schematic illustrating *in situ* formation of a ssDNA tether using a dual-trap optical tweezers. A single dsDNA biotinylated on both ends, but on the same strand, is captured with the optical traps. Then, the non-biotinylated strand is removed by force-induced melting of the duplex. (D) Force-extension curves indicate the transition from dsDNA (black) to ssDNA (red). (E) Example of a kymograph with HELB trajectories moving upwards. (F) Representative kymograph showing that HELB binds but remain stationary on bare ssDNA in the absence of ATP. (G) Scheme of the Gap-DNA substrate, a dsDNA molecule with a gap of 63 nt and no 5′-flap. (H) HELB requires a 5′-flap to unwind duplex DNA at low force. Representative time-courses of MT experiments with Gap-DNA and 100 nM HELB and 1 mM ATP at 1 pN, show no HELB unwinding activity.

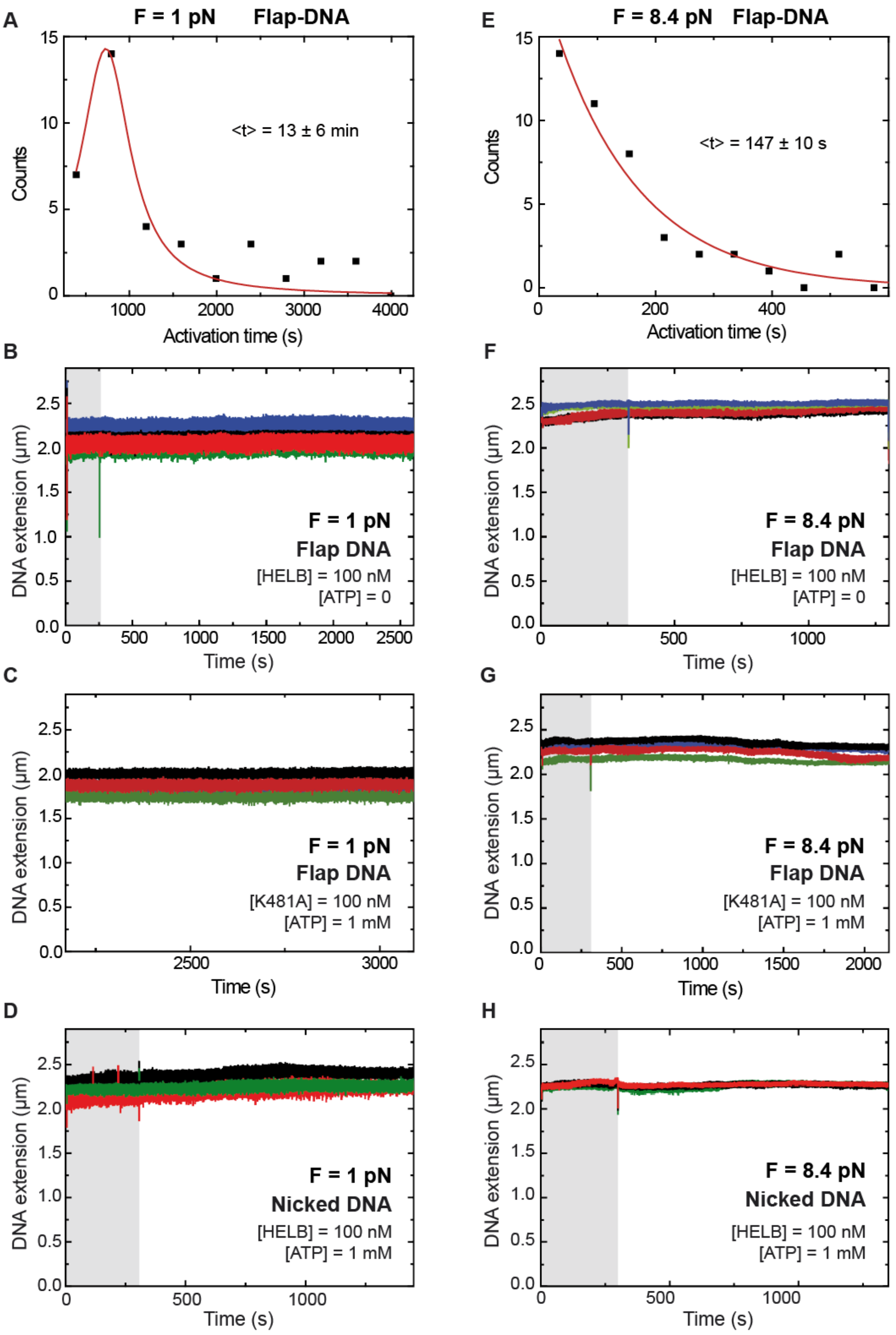

Fig. S3. *Activation time and magnetic tweezers control experiments at 1 pN and 8.4 pN*

(A) Distribution of the activation time, defined as the time until first unwinding event observation, in experiments using Flap-DNA at 1 pN in the presence of 100 nM HELB and 1 mM ATP. The

distribution fits a Lorentzian function centred at 13 ± 6 min (n = 37). (B) Representative time-courses of Flap-DNA control experiments performed in the absence of ATP at 1 pN force. (C) Representative time-courses of Flap-DNA control experiments performed in the presence of 100 nM HELB ATPase mutant at 1 pN. (D) Representative time-courses of experiments employing nicked DNA, done at 1 pN. (E) Distribution of the activation time in experiments using Flap-DNA at 8.4 pN in the presence of 100 nM HELB and 1 mM ATP. The distribution decays exponentially governed by a mean time <t> = 147 ± 10 s (n = 44). (F) Representative time-courses of Flap-DNA control experiments performed in the absence of ATP at 8.4 pN force. (G) Representative time-courses of Flap-DNA control experiments performed in the presence of 100 nM HELB ATPase mutant at 8.4 pN. (H) Representative time-courses of experiments employing nicked DNA, done at 8.4 pN. Shadowed regions indicate the time window of injection of protein’s mixture.

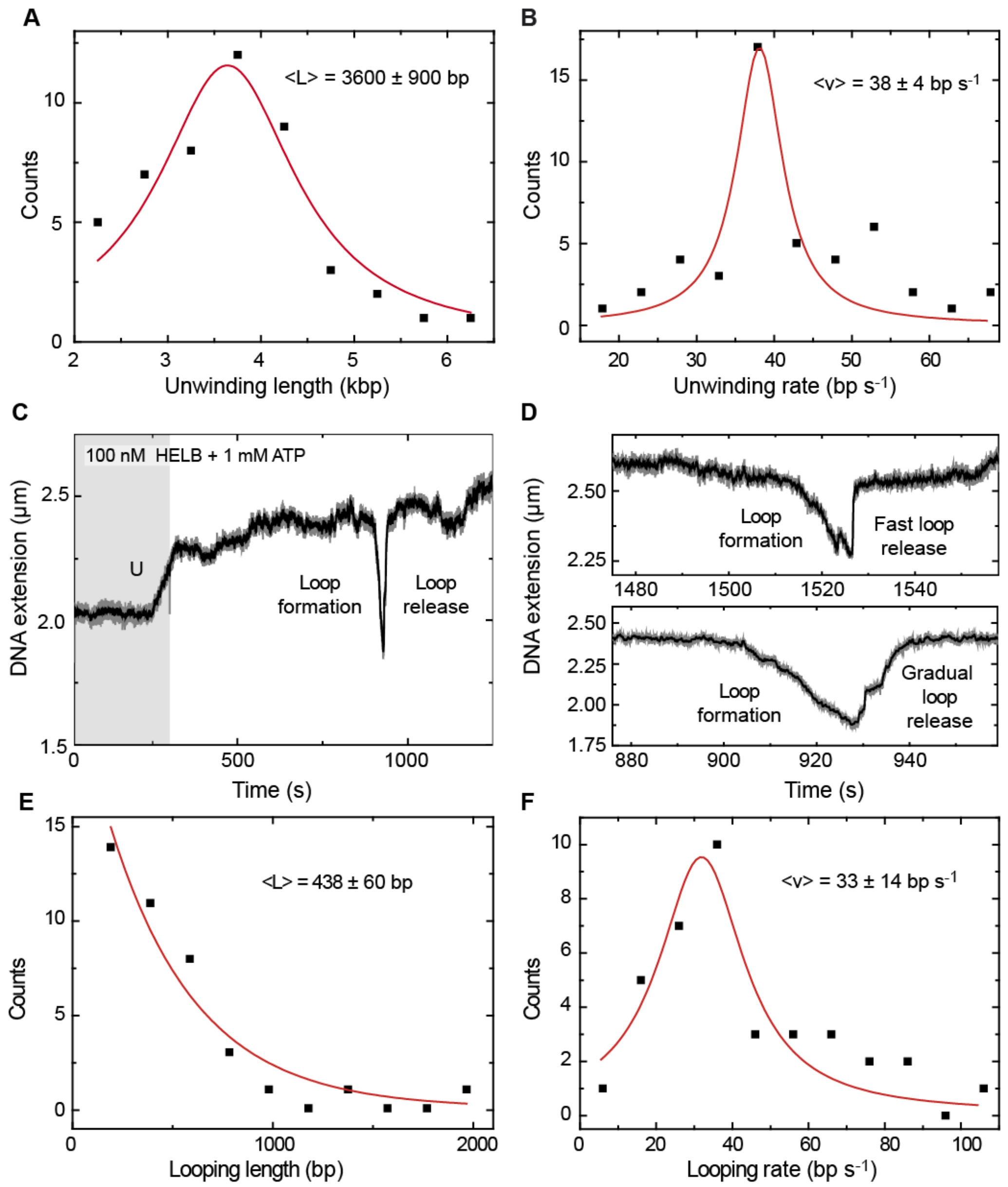

Fig. S4. *Dynamics of DNA loop formation by HELB at high force*

(A) Distribution of the unwinding length of events fitted to a Lorentzian function (red) with a mean unwinding length of <L> = 3600 ± 900 bp (n = 48) measured in MT time-courses. (B) Distribution of the unwinding rate of events fitted to a Lorentzian function with a peak at 38 ± 4 bp s^{-1} (n = 47). (C) Representative unwinding time-course showing a loop that proceeds below the original extension of the tether. (D) Examples of height recovery following looping by a sudden jump in bead's height (upper panel) and gradual recovery of bead's initial position after looping (lower panel). (E) The distribution of looping lengths decays exponentially with a mean length of 438 ± 60 bp (n = 40). (F) Distribution of the looping rate of events, including a Lorentzian function fit with mean velocity of 33 ± 14 bp s^{-1} (n = 37).

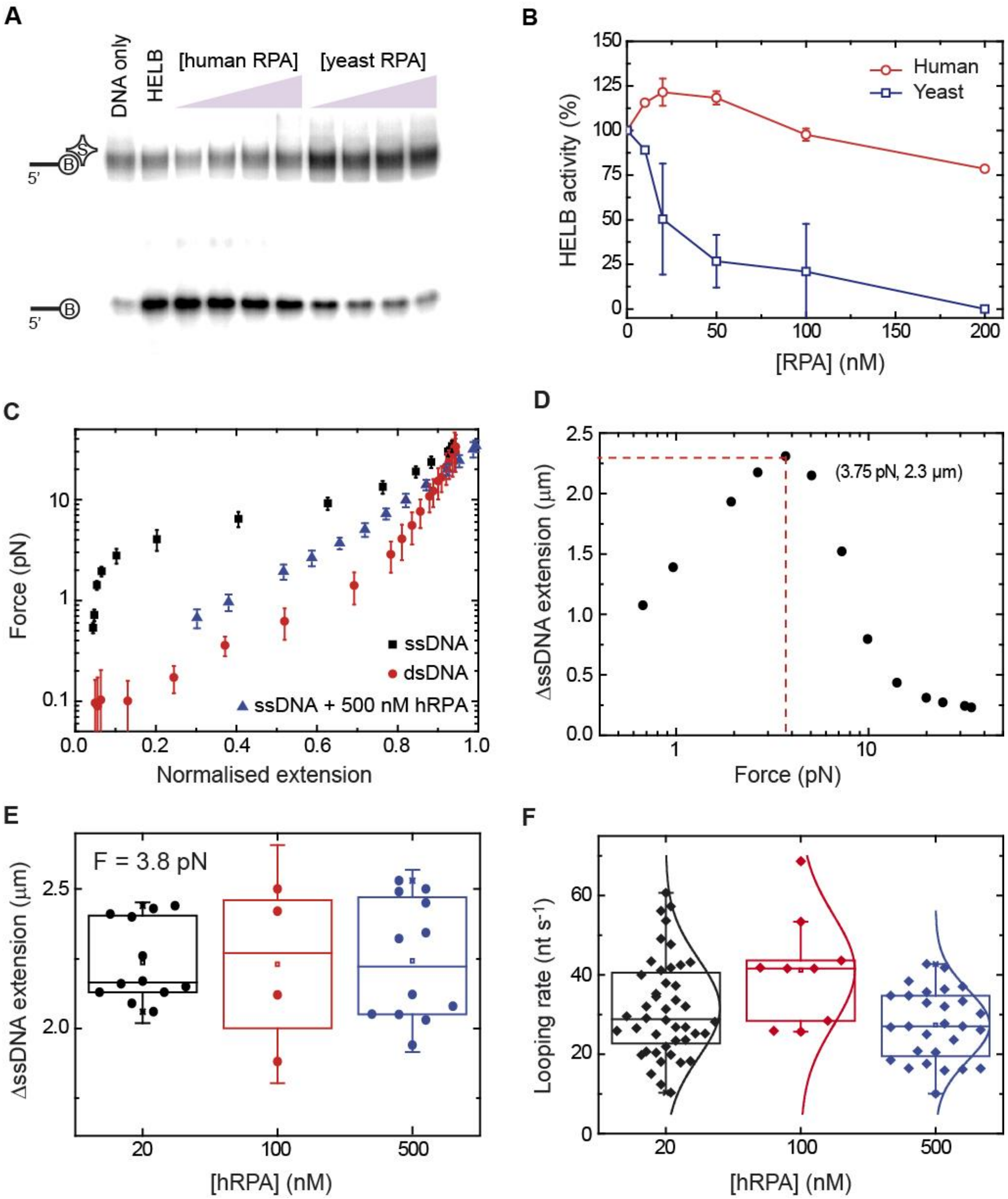

Fig. S5. *HELB ssDNA-dependent activities are RPA species-specific*

(A) Bulk translocase assay based on displacement of streptavidin from 3′-biotinylated oligonucleotides. A time point was chosen (5 mins) where the reaction is not quite complete to be sensitive to changes induced by the presence of RPA. Increasing amounts of either human or yeast RPA were titrated to establish their effect on the translocation of HELB. (B) Quantification of the gel-based assay. HELB translocates at least as efficiently on ssDNA coated with human RPA. Under the same conditions, yeast RPA causes the activity to decrease significantly. (C) Normalised mean force vs. extension curves for ssDNA (black, n = 14), dsDNA (red, n = 12) and ssDNA in the presence of 500 nM hRPA (blue, n = 14) obtained with MT in HELB buffer. (D) Increment of ssDNA extension caused by 500 nM hRPA as a function of the applied force. (E) Change of ssDNA extension due to hRPA binding as a function of hRPA concentration at a constant force of 3.8 pN. (F) HELB looping rate measured on hRPA-covered ssDNA at different hRPA concentrations.

Experiments were performed at 5 nM HELB and 2 mM ATP. Box plots in E and F indicate the mean, median, 25th and 75th percentiles of the distributions and the whiskers show the standard deviation. Solid lines in F are normal distribution fits to the data.

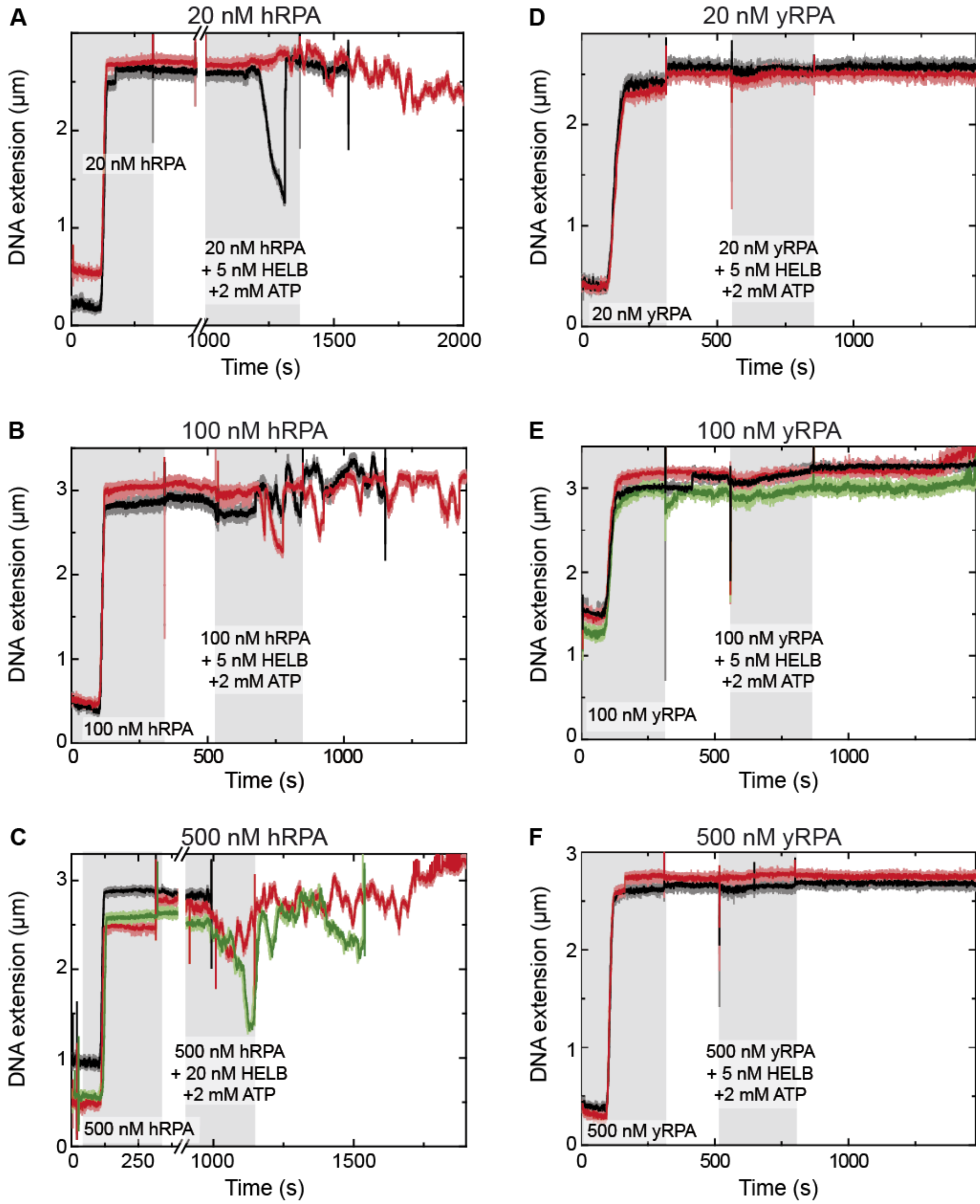

Fig. S6. *Magnetic tweezers experiments show no HELB activity on yeast RPA coated ssDNA*

(A-C) Representative time-courses of MT experiments of HELB on human RPA-coated ssDNA. ssDNA tethers are first exposed to 20 nM, 100 nM or 500 nM hRPA, as indicated. Then, 5 nM or 20 nM HELB and 2 mM ATP are introduced into the fluid cell. Shaded regions indicate the time window of reagents flow. Force is kept constant at 3.8 pN throughout the experiment. Characteristic loop formation and shrinkage dynamics caused by HELB are observed at all RPA concentrations tested. (D-F) Analogous MT experiments to study the activity of HELB on yeast RPA-coated ssDNA. Experimental conditions were as in A-C but using 20 nM, 100 nM or 500 nM yRPA. No HELB activity is detected in the presence of yRPA. Force is kept constant at 3.8 pN throughout the experiments.

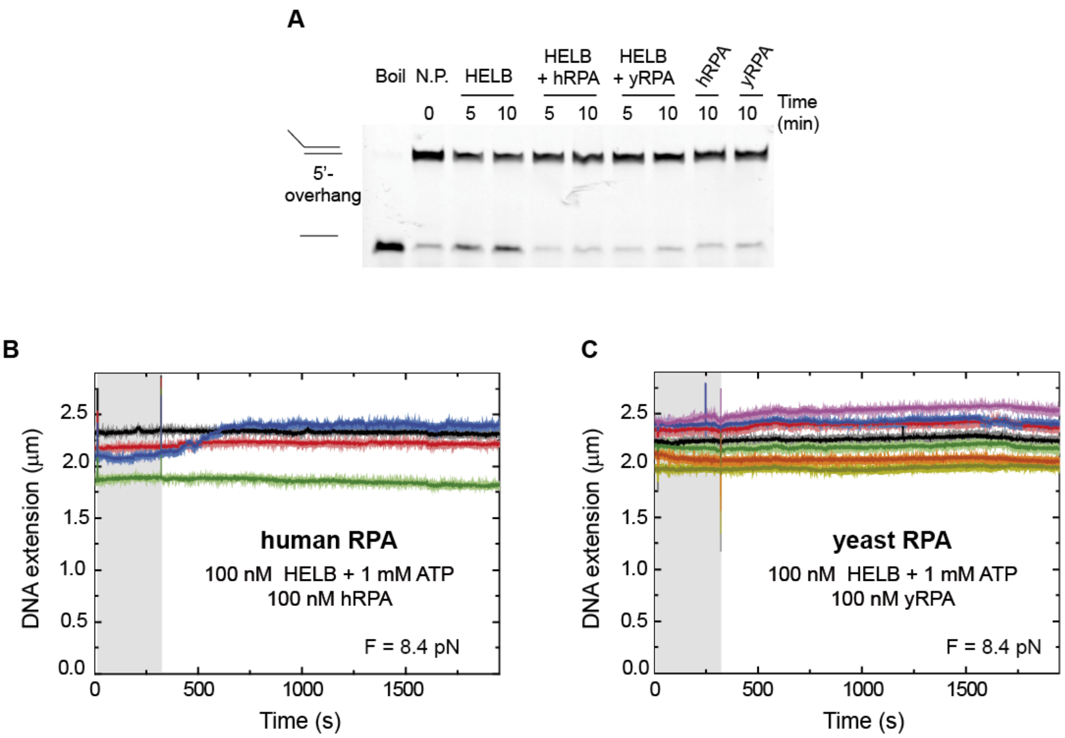

Fig. S7. *RPA hinders HELB unwinding activity in bulk and in single-molecule experiments.*

(A) RPA hinders HELB unwinding activity in bulk. HELB duplex-unwinding activity is fairly poor and appears to be inhibited by RPA. (B) Representative time-courses of MT experiments using Flap-DNA stretched at 8.4 pN and 100 nM HELB, 100 nM human RPA and 1 mM ATP. (C) Similar experiment as described in B but including 100 nM yeast RPA.

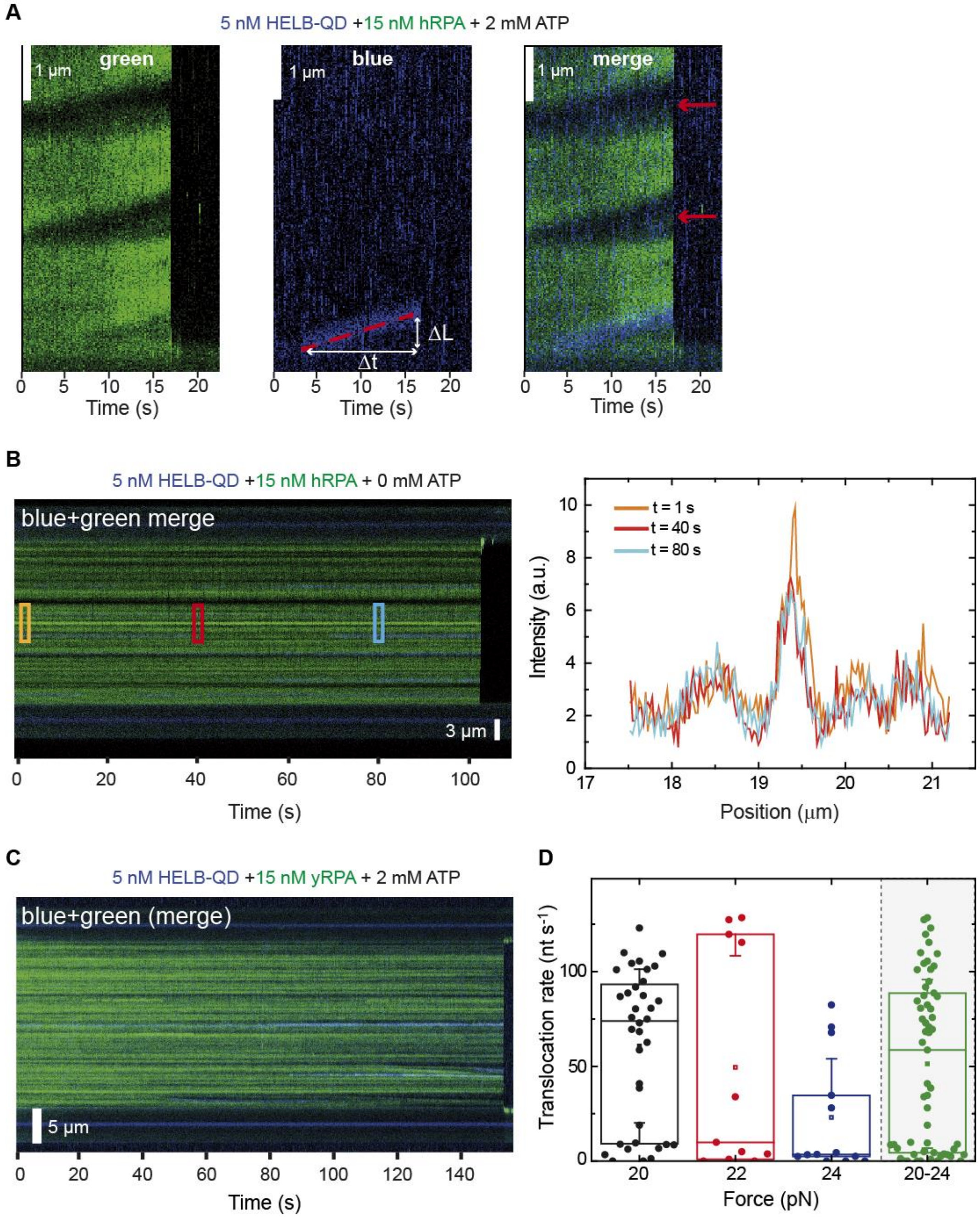

Fig. S8. *HELB displacement of RPA is ATP- and species-specific*

(A) Representative kymograph showing movement of HELB-QD (blue) and removal of hRPA^{MB543} (green) under ATP conditions (F = 15 pN). A schematic is overlaid showing the methodology to calculate HELB translocation rate (ΔL/Δt). Two red arrows in merge panel point to two presumed non-labelled HELB trajectories. (B) (left panel) Representative merge kymograph showing HELB (blue) and ssDNA covered by hRPA^{MB543} (green) in the absence of ATP. HELB binds but remains still in the absence of ATP (F = 19 pN). (right panel) Intensity profiles of the RPA^{MB543} distribution at different times along the trajectory outlined by the coloured rectangles in the merge kymograph. Each line represents the average intensity over 10 frames (1.56 s). No change in the RPA distribution is detected. (C) Representative merge kymograph showing HELB (blue) and a ssDNA

covered by yeast RPA^{CY3} (green) in the presence of ATP. (D) Distribution of HELB translocation rates on yRPA-covered ssDNA (n = 59). HELB translocation on ssDNA is mostly hindered by yeast RPA.

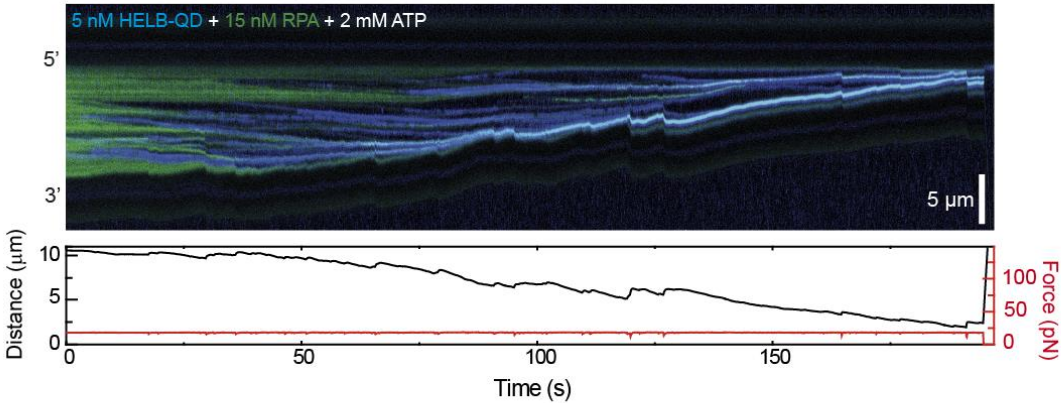

Fig. S9. *Direct observation of loop formation by HELB ssDNA covered by hRPA*

Representative kymograph of activity including HELB-QD (blue) and hRPA (green) in a force-clamp experiment with 5' fixed bead configuration. Measurement of the extension of tether is shown below the kymograph. HELB displaces hRPA and compacts the DNA via formation of loops.

Supplementary Tables

Table S1. *Oligonucleotides used for fabrication of magnetic tweezers substrates*.

Fragment	Oligonucleotide	Sequence
Handles	42.FMH_F2_KpnI-PsiI-ScaI	GCG TAA GTG GTA CCT TAT AAA GTA CTC GAC TCA CTA TAG GGA GAC CGG C
	JOE-R1	AGT AAG CGC CGT CAG ACC AG
Flap	Poly(dT) flap	TTT TTT TTT TTT TTT TTT TTT TTT TTT TTT TTT TTT TTC AGC TAG CCT CAG CCT ACA ATC ACC
Complementary to released BbvCI fragment 1	137.block piece1 BbvCI	GGA TGA CAT GAG CTG A
Complementary to released BbvCI fragment2	138.block piece2 BbvCI	GGG TCA AGT GTG CTG A
Complementary to released BbvCI fragment3	139.block piece3 BbvCI	GGC TAG CTG AGC TGA
Complementary to released BbvCI fragment4	140.block piece4 BbvCI	GGT GAT TGT AGG CTG A

Table S2. *Oligonucleotides used in translocase and helicase assays.*

Assay	Oligonucleotide	Sequence
Helicase assay	3′ substrate	Cy5-GCT TGC TAG GAC GGA TCG CTC GAG GTT TTT TTT TTT TTT TTT TTT<br>+<br>C CTC GAG CGA TCC GTC CTA GCA AGC
	5′ substrate	TTT TTT TTT TTT TTT TTT TTC CTC GAG CGA TCC GTC CTA GCA AGC<br>+<br>Cy5-GCT TGC TAG GAC GGA TCG CTC GAG G
Traslocation assay	5′-3′	5′ - ^{32}P G(biodT)ACGTATTCAAGATACCTCGTACTCTGTACTGACTGATCCTAGG
	3′-5′	5′ - ^{32}P GTACGTATTCAAGATACCTCGTACTCTGTACTGACTCGGATCC(biodT)A
EMSA assay		Cy5-GCT TGC TAG GAC GGA TCG CTC GAG G (25mer) and<br>Cy5-GCT TGC TAG GAC GGA TCG CTC GAG GTT TTT TTT TTT TTT TTT TTT (45mer)

PIFE assay		3'-Cy3 labelled 15mer, 20mer, 30mer, 40mer poly(dT)

Table S3. *Fitting parameters of ATPase assay*

	$$y = \frac{B_{max} \cdot x}{K_d + x}$$	
**Substrate**	**B_{max} (ATP s^{-1})**	**K_d (μM)**
66mer	10.8±0.3	43±4
70mer poly(dT)	20.1±0.5	6.0±0.7
φX174	11.9±0.3	17±1
φX174-RPA	24±1	7±1
Poly(dT)	21.9±0.9	0.7±0.1
Poly(dT)-RPA	35±1	1.3±0.2

Table S4. *Sequence of DNA fragments used in this work*

Underlined sequence: 63 nucleotide gap created after digestion with the nicking enzyme Nt.BbvCI followed by denaturation. Sequence present in Flap-DNA substrate and Gap-DNA substrate.
Red underlined sequence: position of the Poly(dT) flap.
Black underlined sequence: position where the Poly(dT)-flap oligonucleotide anneals.

Fragment	Size (bp)	Sequence
Central part of Flap-DNA substrate, Gap-DNA substrate and torsionally-constrained DNA substrates used in MT experiments.	6337	CAGTTCAGGAAGCGGTGATGCTGATAGAAGCCGGACTGAGTACCTACGAGAAAG<br>AGTGCGCAAAACGCGGTGACGACTATCAGGAAATTTTTGCCCAGCAGGTCCGTG<br>AAACGATGGAGCGCCGTGCAGCCGGTCTTAAACCGCCCGCCTGGGCGGCTGCAG<br>CATTTGAATCCGGGCTGCGACAATCAACAGAGGAGGAGAAGAGTGACAGCAGAG<br>CTGCGTAATCTCCCGCATATTGCCAGCATGGCCTTTAATGAGCCGCTGATGCTT<br>GAACCCGCCTATGCGCGGGTTTTCTTTTGTGCGCTTGCAGGCCAGCTTGGGATC<br>AGCAGCCTGACGGATGCGGTGTCCGGCGACAGCCTGACTGCCCAGGAGGCACTC<br>GCGACGCTGGCATTATCCGGTGATGATGACGGACCACGACAGGCCCGCAGTTAT<br>CAGGTCATGAACGGCATCGCCGTGCTGCCGGTGTCCGGCACGCTGGTCAGCCGG<br>ACGCGGGCGCTGCAGCCGTACTCGGGGATGACCGGTTACAACGGCATTATCGCC<br>CGTCTGCAACAGGCTGCCAGCGATCCGATGGTGGACGGCATTCTGCTCGATATG<br>GACACGCCCGGCGGGATGGTGGCGGGGGCATTTGACTGCGCTGACATCATCGCC<br>CGTGTGCGTGACATAAAACCGGTATGGGCGCTTGCCAACGACATGAACTGCAGT<br>GCAGGTCAGTTGCTTGCCAGTGCCGCCTCCCGGCGTCTGGTCACGCAGACCGCC<br>CGGACAGGCTCCATCGGCGTCATGATGGCTCACAGTAATTACGGTGCTGCGCTG<br>GAGAAACAGGGTGTGGAAATCACGCTGATTTACAGCGGCAGCCATAAGGTGGAT<br>GGCAACCCCTACAGCCATCTTCCGGATGACGTCCGGGAGACACTGCAGTCCCGG<br>ATGGACGCAACCCGCCAGATGTTTGCGCAGAAGGTGTCGGCATATACCGGCCTG<br>TCCGTGCAGGTTGTGCTGGATACCGAGGCTGCAGTGTACAGCGGTCAGGAGGCC<br>ATTGATGCCGGACTGGCTGATGAACTTGTTAACAGCACCGATGCGATCACCGTC<br>ATGCGTGATGCACTGGATGCACGTAAATCCCGTCTCTCAGGAGGGCGAATGACC<br>AAAGAGACTCAATCAACAACTGTTTCAGCCACTGCTTCGCAGGCTGACGTTACT<br>GACGTGGTGCCAGCGACGGAGGGCGAGAACGCCAGCGCGGCGCAGCCGGACGTG<br>AACGCGCAGATCACCGCAGCGGTTGCGGCAGAAAACAGCCGCATTATGGGGATC<br>CTCAACTGTGAGGAGGCTCACGGACGCGAAGAACAGGCACGCGTGCTGGCAGAA

		ACCCCCGGTATGACCGTGAAAACGGCCCGCCGCATTCTGGCCGCAGCACCACAG<br>AGTGCACAGGCGCGCAGTGACACTGCGCTGGATCGTCTGATGCAGGGGGCACCG<br>GCACCGCTGGCTGCAGGTAACCCGGCATCTGATGCCGTTAACGATTTGCTGAAC<br>ACACCAGTGTAAGGGATGTTTATGACGAGCAAAGAAACCTTTACCCATTACCAG<br>CCGCAGGGCAACAGTGACCCGGCTCATACCGCAACCGCGCCCGGCGGATTGAGT<br>GCGAAAGCGCCTGCAATGACCCCGCTGATGCTGGACACCTCCAGCCGTAAGCTG<br>GTTGCGTGGGATGGCACCACCGACGGTGCTGCCGTTGGCATTCTTGCGGTTGCT<br>GCTCGAGCCTCAGCTCATGTCATCCTCAGCACACTTGACCCTCAGCTCAGCTAG<br>CCTCAGCCTACAATCACCTCAGCGAATTCGGTGACCCTTACGCGAATCCGCTTT<br>CAGACGTTGACTGGTCGCGTCTGGCAAAAGTTAAAGACCTGACGCCCGGCGAAC<br>TGACCGCTGAGTCCTATGACGACAGCTATCTCGATGATGAAGATGCAGACTGGA<br>CTGCGACCGGGCAGGGGCAGAAATCTGCCGGAGATACCAGCTTCACGCTGGCGT<br>GGATGCCCGGAGAGCAGGGGCAGCAGGCGCTGCTGGCGTGGTTTAATGAAGGCG<br>ATACCCGTGCCTATAAAATCCGCTTCCCGAACGGCACGGTCGATGTGTTCCGTG<br>GCTGGGTCAGCAGTATCGGTAAGGCGGTGACGGCGAAGGAAGTGATCACCCGCA<br>CGGTGAAAGTCACCAATGTGGGACGTCCGTCGATGGCAGAAGATCGCAGCACGG<br>TAACAGCGGCAACCGGCATGACCGTGACGCCTGCCAGCACCTCGGTGGTGAAAG<br>GGCAGAGCACCACGCTGACCGTGGCCTTCCAGCCGGAGGGCGTAACCGACAAGA<br>GCTTTCGTGCGGTGTCTGCGGATAAAACAAAAGCCACCGTGTCGGTCAGTGGTA<br>TGACCATCACCGTGAACGGCGTTGCTGCAGGCAAGGTCAACATTCCGGTTGTAT<br>CCGGTAATGGTGAGTTTGCTGCGGTTGCAGAAATTACCGTCACCGCCAGTTAAT<br>CCGGAGAGTCAGCGATGTTCCTGAAAACCGAATCATTTGAACATAACGGTGTGA<br>CCGTCACGCTTTCTGAACTGTCAGCCCTGCAGCGCATTGAGCATCTCGCCCTGA<br>TGAAACGGCAGGCAGAACAGGCGGAGTCAGACAGCAACCGGAAGTTTACTGTGG<br>AAGACGCCATCAGAACCGGCGCGTTTCTGGTGGCGATGTCCCTGTGGCATAACC<br>ATCCGCAGAAGACGCAGATGCCGTCCATGAATGAAGCCGTTAAACAGATTGAGC<br>AGGAAGTGCTTACCACCTGGCCCACGGAGGCAATTTCTCATGCTGAAAACGTGG<br>TGTACCGGCTGTCTGGTATGTATGAGTTTGTGGTGAATAATGCCCCTGAACAGA<br>CAGAGGACGCCGGGCCCGCAGAGCCTGTTTCTGCGGGAAAGTGTTCGACGGTGA<br>GCTGAGTTTTGCCCTGAAACTGGCGCGTGAGATGGGGCGACCCGACTGGCGTGC<br>CATGCTTGCCGGGATGTCATCCACGGAGTATGCCGACTGGCACCGCTTTTACAG<br>TACCCATTATTTTCATGATGTTCTGCTGGATATGCACTTTTCCGGGCTGACGTA<br>CACCGTGCTCAGCCTGTTTTTCAGCGATCCGGATATGCATCCGCTGGATTTCAG<br>TCTGCTGAACCGGCGCGAGGCTGACGAAGAGCCTGAAGATGATGTGCTGATGCA<br>GAAAGCGGCAGGGCTTGCCGGAGGTGTCCGCTTTGGCCCGGACGGGAATGAAGT<br>TATCCCCGCTTCCCCGGATGTGGCGGACATGACGGAGGATGACGTAATGCTGAT<br>GACAGTATCAGAAGGGATCGCAGGAGGAGTCCGGTATGGCTGAACCGGTAGGCG<br>ATCTGGTCGTTGATTTGAGTCTGGATGCGGCCAGATTTGACGAGCAGATGGCCA<br>GAGTCAGGCGTCATTTTTCTGGTACGGAAAGTGATGCGAAAAAAACAGCGGCAG<br>TCGTTGAACAGTCGCTGAGCCGACAGGCGCTGGCTGCACAGAAAGCGGGGATTT<br>CCGTCGGGCAGTATAAAGCCGCCATGCGTATGCTGCCTGCACAGTTCACCGACG<br>TGGCCACGCAGCTTGCAGGCGGGCAAAGTCCGTGGCTGATCCTGCTGCAACAGG<br>GGGGGCAGGTGAAGGACTCCTTCGGCGGGATGATCCCCATGTTCAGGGGGCTTG<br>CCGGTGCGATCACCCTGCCGATGGTGGGGGCCACCTCGCTGGCGGTGGCGACCG<br>GTGCGCTGGCGTATGCCTGGTATCAGGGCAACTCAACCCTGTCCGATTTCAACA<br>AAACGCTGGTCCTTTCCGGCAATCAGGCGGGACTGACGGCAGATCGTATGCTGG<br>TCCTGTCCAGAGCCGGGCAGGCGGCAGGGCTGACGTTTAACCAGACCAGCGAGT<br>CACTCAGCGCACTGGTTAAGGCGGGGGTAAGCGGTGAGGCTCAGATTGCGTCCA<br>TCAGCCAGAGTGTGGCGCGTTTCTCCTCTGCATCCGGCGTGGAGGTGGACAAGG<br>TCGCTGAAGCCTCTAGAGAATGCTACGTACCTGATGAGCTCCAGCTTTTGTTCC<br>CTTTAGTGAGGGTTAATTGCGCGCTTGGCGTAATCATGGTCATAGCTGTTTCCT<br>GTGTGAAATTGTTATCCGCTCACAATTCCACACAACATACGAGCCGGAAGCATA<br>AAGTGTAAAGCCTGGGGTGCCTAATGAGTGAGCTAACTCACATTAATTGCGTTG<br>CGCTCACTGCCCGCTTTCCAGTCGGGAAACCTGTCGTGCCAGCTGCATTAATGA<br>ATCGGCCAACGCGCGGGGAGAGGCGGTTTGCGTATTGGGCGCTCTTCCGCTTCC<br>TCGCTCACTGACTCGCTGCGCTCGGTCGTTCGGCTGCGGCGAGCGGTATCAGCT<br>CACTCAAAGGCGGTAATACGGTTATCCACAGAATCAGGGGATAACGCAGGAAAG<br>AACATGTGAGCAAAAGGCCAGCAAAAGGCCAGGAACCGTAAAAAGGCCGCGTTG<br>CTGGCGTTTTTCCATAGGCTCCGCCCCCCTGACGAGCATCACAAAAATCGACGC<br>TCAAGTCAGAGGTGGCGAAACCCGACAGGACTATAAAGATACCAGGCGTTTCCC<br>CCTGGAAGCTCCCTCGTGCGCTCTCCTGTTCCGACCCTGCCGCTTACCGGATAC<br>CTGTCCGCCTTTCTCCCTTCGGGAAGCGTGGCGCTTTCTCATAGCTCACGCTGT<br>AGGTATCTCAGTTCGGTGTAGGTCGTTCGCTCCAAGCTGGGCTGTGTGCACGAA

		CCCCCCGTTCAGCCCGACCGCTGCGCCTTATCCGGTAACTATCGTCTTGAGTCC<br>AACCCGGTAAGACACGACTTATCGCCACTGGCAGCAGCCACTGGTAACAGGATT<br>AGCAGAGCGAGGTATGTAGGCGGTGCTACAGAGTTCTTGAAGTGGTGGCCTAAC<br>TACGGCTACACTAGAAGGACAGTATTTGGTATCTGCGCTCTGCTGAAGCCAGTT<br>ACCTTCGGAAAAAGAGTTGGTAGCTCTTGATCCGGCAAACAAACCACCGCTGGT<br>AGCGGTGGTTTTTTTGTTTGCAAGCAGCAGATTACGCGCAGAAAAAAAGGATCT<br>CAAGAAGATCCTTTGATCTTTTCTACGGGGTCTGACGCTCAGTGGAACGAAAAC<br>TCACGTTAAGGGATTTTGGTCATGAGATTATCAAAAAGGATCTTCACCTAGATC<br>CTTTTAAATTAAAAATGAAGTTTTAAATCAATCTAAAGTATATATGAGTAAACT<br>TGGTCTGACAGTTACCAATGCTTAATCAGTGAGGCACCTATCTCAGCGATCTGT<br>CTATTTCGTTCATCCATAGTTGCCTGACTCCCCGTCGTGTAGATAACTACGATA<br>CGGGAGGGCTTACCATCTGGCCCCAGTGCTGCAATGATACCGCGAGACCCACGC<br>TCACCGGCTCCAGATTTATCAGCAATAAACCAGCCAGCCGGAAGGGCCGAGCGC<br>AGAAGTGGTCCTGCAACTTTATCCGCCTCCATCCAGTCTATTAATTGTTGCCGG<br>GAAGCTAGAGTAAGTAGTTCGCCAGTTAATAGTTTGCGCAACGTTGTTGCCATT<br>GCTACAGGCATCGTGGTGTCACGCTCGTCGTTTGGTATGGCTTCATTCAGCTCC<br>GGTTCCCAACGATCAAGGCGAGTTACATGATCCCCCATGTTGTGCAAAAAAGCG<br>GTTAGCTCCTTCGGTCCTCCGATCGTTGTCAGAAGTAAGTTGGCCGCAGTGTTA<br>TCACTCATGGTTATGGCAGCACTGCATAATTCTCTTACTGTCATGCCATCCGTA<br>AGATGCTTTTCTGTGACTGGTGAGTACTCAACCAAGTCATTCTGAGAATAGTGT<br>ATGCGGCGACCGAGTTGCTCTTGCCCGGCGTCAATACGGGATAATACCGCGCCA<br>CATAGCAGAACTTTAAAAGTGCTCATCATTGGAAAACGTTCTTCGGGGCGAAAA<br>CTCTCAAGGATCTTACCGCTGTTGAGATCCAGTTCGATGTAACCCACTCGTGCA<br>CCCAACTGATCTTCAGCATCTTTTACTTTCACCAGCGTTTCTGGGTGAGCAAAA<br>ACAGGAAGGCAAAATGCCGCAAAAAAGGGAATAAGGGCGACACGGAAATGTTGA<br>ATACTCATACTCTTCCTTTTTCAATATTATTGAAGCATTTATCAGGGTTATTGT<br>CTCATGAGCGGATACATATTTGAATGTATTTAGAAAAATAAACAAATAGGGGTT<br>CCGCGCACATTTCCCCGAAAAGTGCCACCTAAATTGTAAGCGTTAATATTTTGT<br>TAAAATTCGCGTTAAATTTTTGTTAAATCAGCTCATTTTTTAACCAATAGGCCG<br>AAATCGGCAAAATCCCTTA

Movie S1 (separate file). *HELB translocation on ssDNA and RPA displacement*

Video of a ssDNA tether between two optically-trapped beads covered by green fluorescent human RPA^{MB543} (15 nM) showing the activity of biotinylated HELB labelled with QDs (5 nM HELB and 2 mM ATP, blue emission). The translocation of HELB towards the left bead is coupled to the displacement of RPA. F = 18 pN, pixel size = 50 nm, 5 fps.
